# Synthesis of substituted *Z*-styrenes by Hiyama-type coupling of oxasilacycloalkenes: application to the synthesis of a 1-benzoxocane

**DOI:** 10.3762/bjoc.13.209

**Published:** 2017-10-11

**Authors:** James R Vyvyan, Courtney A Engles, Scott L Bray, Erik D Wold, Christopher L Porter, Mikhail O Konev

**Affiliations:** 1 Department of Chemistry, Western Washington University, 516 High Street, Bellingham, WA 98225, USA

**Keywords:** cross-coupling, heterocycles, hydrosilylation, palladium, silicon

## Abstract

Several Hiyama cross-coupling reactions of oxasilacycloalkenes and aryl iodides are described that produce trisubstituted *Z*-styrenes in moderate to excellent yields. Both electron-rich and electron-poor aryl iodides are tolerated in the cross-coupling reaction. The oxasilacycloalkene coupling partners were prepared by ruthenium-catalyzed intramolecular anti-hydrosilylation of alkynols. One of the cross-coupling products was converted to a 1-benzoxocane, albeit in low yield, using an intramolecular Buchwald–Hartwig etherification. The cyclic ether produced contains the carbon skeleton of heliannuol A.

## Introduction

**Figure 1:**
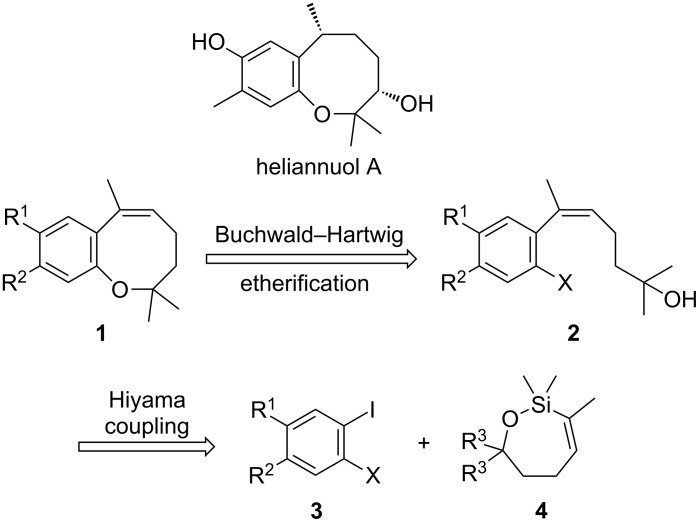
Retrosynthetic analysis of heliannuol A.

The development of transition metal-catalyzed cross-coupling technologies over the last four decades revolutionized the synthetic chemistry. Indeed, the importance of Pd-catalyzed coupling was recognized with the 2010 Nobel Prize awarded to Heck, Negishi and Suzuki [1–2]. Developed somewhat later than the aforementioned methods and the couplings of organomagnesium and organotin reagents was the cross-coupling of silanes, pioneered by Hiyama [3–4] and siloxanes [5]. This versatile method has been extensively reviewed [6–8]. The cross-coupling of alkenyl silanols [9–10] and oxasilacycloalkenes (cyclic siloxanes, cf. Figure 1) [6–8] is an excellent method to prepare stereodefined alkenes. The cyclic siloxanes can be prepared in a number of ways: hydrosilylation of alkynes [11–13], semihydrogenation of silyl alkynes [14], ring-closing metathesis (RCM) [15–21] and enyne metathesis [22–23].

We became interested in the cross-coupling of cyclic siloxanes in the context of preparing trisubstituted *Z*-styrenes for the synthesis of natural product targets [24]. Heliannuol A was the first member of a family of allelopathic [25–27] sesquiterpenoids isolated from the sunflower *Helianthus annuus* [28–33], and it contains an unusual benzoxocane moiety. Owing to their unusual structures and biological activity, the heliannuols have attracted significant attention from synthetic chemists. The various approaches to the heliannuols have recently been reviewed [34]. We have previously reported syntheses of heliannuols C, D, and E via intramolecular epoxide opening reactions [35–36], but this approach did not translate well to the synthesis of heliannuol A. One of our alternative strategies for the synthesis of heliannuol A was an intramolecular Buchwald–Hartwig etherification of a *Z*-styrene derivative to provide a conformational constraint to facilitate the formation of the eight-membered ring [37]. The presence of the alkene reduces the conformational degrees of freedom in **2**, thereby partially offsetting the entropic penalty of forming the eight-membered ring. We endeavored to test the strategy through the preparation of a simplified model compound **1** (Figure 1). We envisaged the precursor to the cycloetherification, **2**, would be prepared from the Hiyama-type cross-coupling of the appropriate aryl iodide **3** and the oxasilacycloalkene **4**.

## Results and Discussion

**Scheme 1:**
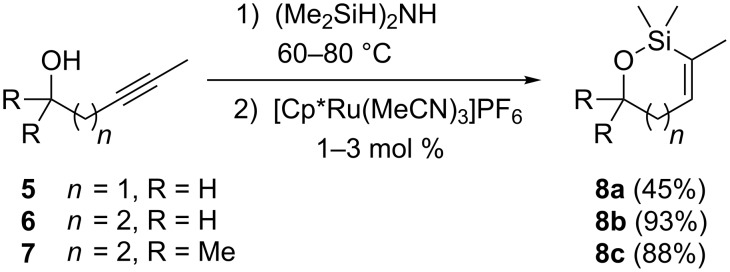
Hydrosilylation of alkynols.

The oxasilacycloalkenes used in this study were readily prepared by intramolecular anti-hydrosilylation of alkynols **5**–**7** using the method reported by Ball and Trost (Scheme 1) [12–13]. Alkynol **5** was commercially available, **6** was readily prepared following literature procedures (see Experimental section), and alkynol **7** was, surprisingly, a new compound. The hydrosilylation reactions of **5**–**7** can be carried out on gram scales and generally produce high yields of the siloxane products after distillation. Siloxane **8a** was isolated in more modest yield, however, probably owing to its volatility.

The siloxanes **8** participated efficiently in Hiyama-type cross-couplings with aryl iodides in the presence of Pd_2_(dba)_3_ catalyst and tetrabutylammonium fluoride (TBAF, Table 1). Both electron-rich (Table 1, entries 1, 2, and 7) and electron-poor (Table 1, entries 5 and 6) iodides give coupled products in moderate to excellent yields. When multiple halogens were present, high selectivity for reaction at the aryl iodide was observed (Table 1, entries 3–5). Pyridyl iodide **22** also worked well in the cross-coupling (Table 1, entry 8).

**Table 1:** Pd-catalyzed couplings of oxasilacycloalkenes with aryl iodides.

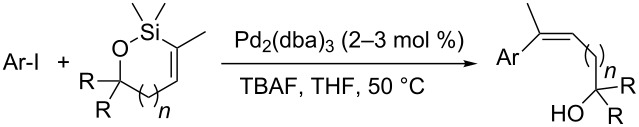				

Entry	Aryl iodide	Siloxane	Product	Yield (%)

1	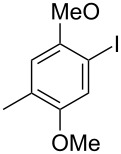 **9**	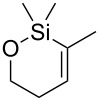 **8a**	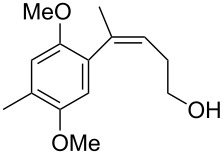 **10**	92^a^
2	**9**	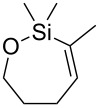 **8b**	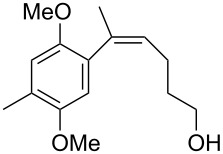 **11**	52
3	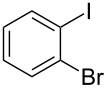 **12**	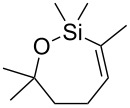 **8c**	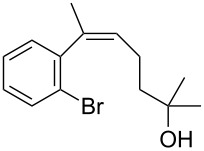 **13**	87
4	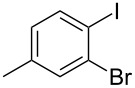 **14**	**8c**	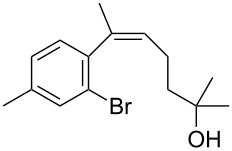 **15**	94
5	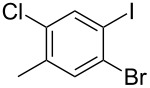 **16**	**8c**	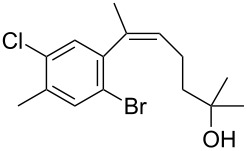 **17**	80
6	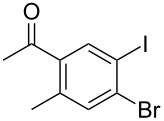 **18**	**8c**	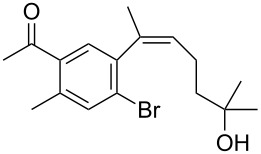 **19**	60
7	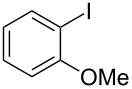 **20**	**8c**	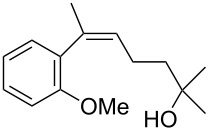 **21**	48
8	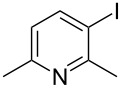 **22**	**8c**	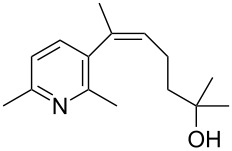 **23**	58

^a^10 mol % Pd_2_(dba)_3_ used in this trial.

Having prepared a number of substituted *Z*-styrenes, we next focused on the intramolecular Buchwald–Hartwig etherification [38–40] of bromoalcohol **15** to prepare eight-membered cyclic ether **24** (Table 2). The use of Pd(II) catalyst precursors with BINAP ligands and carbonate bases in toluene [38–39] was ineffective, returning significant amounts of unreacted starting material (Table 2, entry 1) or debrominated material **25** (Table 2, entry 2). The use of a bis(diphenylphosphino)ferrocenyl (dppf) ligand and a stronger base to irreversibly deprotonate the alcohol was similarly ineffective in both toluene and dioxane (Table 2, entry 3). Based on Hartwig’s success with similar intramolecular etherifications to make five- and six-membered rings [40], and our own preparation of an analogous seven-membered cyclic ether [41], we examined Pd(0) catalyst precursors with the Q-Phos ligand. The reaction with Pd(dba)_2_ with Q-Phos and sodium *tert*-butoxide produced cyclic ether **24** along with debrominated **25** as the major product (Table 2, entry 4). As before, irreversibly deprotonating the alcohol with sodium hydride was not productive (Table 2, entry 5). Using Pd_2_(dba)_3_ as catalyst precursor increased the yield of **24** relative to the amount of **25** produced, but the overall yield of the cyclic ether was still low (Table 2, entry 6). Microwave heating was not helpful (Table 2, entry 7). The non-nucleophilic base DBU was not effective in promoting the cyclization, either (Table 2, entry 8). We observed small amounts of products arising from the dehydration of **15** in the ^1^H NMR spectra of the crude reaction mixtures from these experiments, so we were reluctant to increase the reaction temperature further.

**Table 2:** Buchwald–Hartwig etherifications of bromoalcohol **15**.

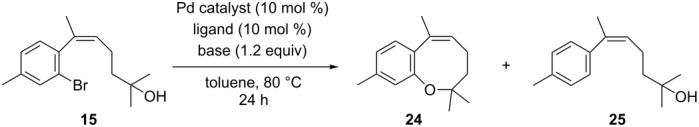					

Entry	Pd catalyst	Ligand	Base	Yield(%)	
				**24**	**25**

1	Pd(OAc)_2_	tol-BINAP	K_2_CO_3_	0	0^a^
2	Pd(TFA)_2_	tol-BINAP	Cs_2_CO_3_	0	21
3	Pd(OAc)_2_	dppf	NaH	0	45^b^
4	Pd(dba)_2_	Q-Phos	NaO*t*-Bu	7	53
5	Pd(dba)_2_	Q-Phos	NaH	0	40
6	Pd_2_(dba)_3_	Q-Phos	NaO*t*-Bu	10	12
7^c^	Pd(dba)_2_	Q-Phos	NaO*t*-Bu	6	21^d^
8	Pd(dba)_2_	Q-Phos	DBU	0	0^e^

^a^73% starting material recovered. ^b^Using dioxane as solvent gave a 32% yield of **25**. ^c^Microwave heating, 80 °C, 18 h. ^d^25% starting material recovered. ^e^65% starting material recovered.

We also hydrogenated the conjugated olefin of **15** to increase the flexibility of the tether, in case the conformational constraint was actually hindering the cyclization, but no cyclic ether was observed when this material was subjected to Pd catalyst and base (not shown).

**Scheme 2:**
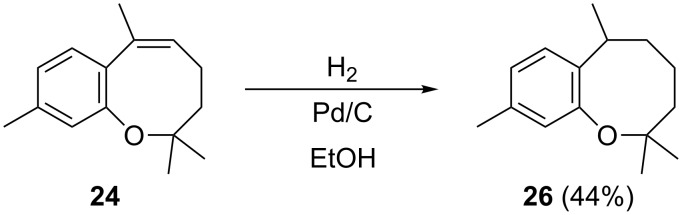
Hydrogenation of benzoxocane **24**.

Hydrogenation of **24** over Pd/C gave **26**, whose ^1^H NMR spectrum matches that of the benzoxocane prepared by Pettus and co-workers [42] (Scheme 2), which was shown by them to be the now-refuted structure of helianane.

## Conclusion

In summary, we have confirmed the Hiyama cross-coupling of cyclic siloxanes is an efficient route to *Z*-trisubstituted styrenes that are useful for the synthesis of natural product frameworks. Although yields are low under the conditions attempted, a proof of concept has been established in applying the Buchwald–Hartwig coupling to **15**, producing the benzoxocane **24**, which contains the carbon skeleton of heliannuol A.

## Supporting Information

File 1 Full experimental details and copies of ^1^H and ^13^C NMR spectra for all new compounds.
