# Supplementary material for: Synthesis of substituted Z-styrenes by Hiyama-type coupling of oxasilacycloalkenes: application to the synthesis of a 1-benzoxocane
Source: Beilstein J Org Chem. 2017 Oct 11;13:2122–7. doi: 10.3762/bjoc.13.209 (PMC5647712; doi:10.3762/bjoc.13.209)

Supporting Information

for

Synthesis of substituted *Z*-styrenes by Hiyama-type coupling of oxasilacycloalkenes: application to the synthesis of a 1-benzoxocane

James R. Vyvyan,* Courtney A. Engles, Scott L. Bray, Erik D. Wold, Christopher L. Porter, Mikhail O. Konev
Address: Department of Chemistry, Western Washington University, 516 High Street, Bellingham, WA 98225, USA
Email: James R. Vyvyan - James.Vyvyan@wwu.edu

*Corresponding author

Full experimental details and copies of ^{1}H and ^{13}C NMR spectra for all new compounds

Contents

Experimental procedures S2–S10
Copies of NMR spectra
Alkynol **7** S11
Siloxane **8a** S12
Siloxane **8b** S13
Siloxane **8c** S14
Alcohol **10** S15
Alcohol **11** S16
Alcohol **13** S17
Alcohol **15** S18
Iodide **16** S19
Alcohol **17** S20
Iodide **18** S21
Alcohol **19** S22
Alcohol **21** S23
Alcohol **23** S24
Ether **24** S25
Alcohol **25** S26
Benzoxocane **26** S27

Experimental

All reactions involving air- or moisture-sensitive materials were done in oven-dried glassware under a dry argon atmosphere. CH_2Cl_2, Et_2O, THF and toluene were dried by passing through a column of activated alumina under nitrogen immediately before use. 1,4-Dioxane was distilled from sodium/benzophenone ketyl under argon. Acetone was dried by stirring over boron oxide overnight followed by distillation. Chromatography was carried out using hand-packed columns of silica gel (230–400 mesh). ^{1}H and ^{13}C NMR spectra were acquired on Varian Mercury (300 MHz) or Inova (500 MHz) spectrometers. ^{1}H NMR chemical shifts are referenced to tetramethylsilane (TMS) at 0.00 ppm. ^{13}C NMR chemical shifts are referenced to $CDCl_3$ at 77.0 ppm.

2-Methyl-5-heptyn-2-ol (**7**). 5-Iodo-2-pentyne[1] (6.00 g, 30.9 mmol) was dissolved in dry Et_2O (300 mL) and the solution was cooled in a dry ice–isopropyl alcohol bath. *tert*-Butyllithium (41.5 mL, 1.64 M in pentane, 68.1 mmol, 2.20 equiv) was added slowly. After 20 min, dry acetone (6.80 mL, 5.38 g, 92.7 mmol, 3.0 equiv) was added dropwise. After warming to room temperature, the white suspension was quenched by careful addition of NH_4Cl solution (~100 mL). The layers were separated and the aqueous layer was extracted with Et_2O (3 × 40 mL). The combined organic layers were washed with sat. NH_4Cl solution and brine. The organic layer was dried over Na_2SO_4, filtered, and concentrated by rotary evaporation. MPLC (3:1 hexanes: EtOAc) gave **7** as a colorless oil (2.04 g, 16.2 mmol, 52%). $R_f = 0.35$ (3:1 hexanes:EtOAc); IR (neat, diamond ATR): 3392 br, 2922, 2857, 2100 w, 1468, 1447, 1377, 1218, 1134, 927, 909 cm^{-1}; ^{1}H NMR ($CDCl_3$, 300 MHz): δ = 2.25 (tq, J = 7.5, 2.5 Hz, 2H), 2.35 (br s, 1H, OH), 1.78 (t, J = 2.5 Hz, 3H), 1.22 (s, 6H); ^{13}C NMR ($CDCl_3$, 75 MHz): δ = 79.2, 76.2, 70.6, 42.0, 28.9, 13.8, 3.3; *Anal.* Calcd for $C_8H_{14}O$: C, 76.14; H, 11.18. Found: C, 76.50; H, 11.35.

[1] Tauber, J.; Rudolph, K.; Rohr, M.; Erkel, G.; Opatz, T. *Eur. J. Org. Chem.*, **2015**, 3587-3608.

Alternative preparation of ***7***

A solution of MeMgBr (23.0 mL, 3.0 M in Et_2O, 69.0 mmol, 1.1 equiv) was diluted with additional Et_2O (23 mL). A solution of 5-heptyn-2-one[2] (6.69 g, 61.0 mmol, 1.0 equiv) in dry Et_2O (10 mL) was added to dropwise through an addition funnel such that the mixture refluxed gently. After 1 h at room temperature, half-saturated NH_4Cl solution was carefully added and the mixture transferred to a separatory funnel. The layers were separated and the aqueous layer extracted with ether (3 × 30 mL). The combined organic layers were washed with sat. NH_4Cl solution and brine. The organic layer was dried over Na_2SO_4, filtered, and concentrated by rotary evaporation. The residue was distilled to provide **7** (5.35 g, 42.4 mmol, 70%) as a colorless oil (bp = 69-72 °C at 9 mmHg). For spectral characterization, *vide supra*.

2,2,3-Trimethyl-5,6-dihydro-2*H*-1,2-oxasiline (8a). A 2-necked 100-mL round-bottomed flask was charged with 3-pentyn-1-ol (**5**) (6.92 mL, 6.23 g, 71.0 mmol) and 1,1,3,3-tetramethyldisilazane (26.6 mL, 20.0 g, 157 mmol). The solution was heated in an 80 °C oil bath for 2.5 h. The flask was removed from the oil bath and the excess 1,1,3,3-tetramethyldisilazane was removed by distillation. The residue was taken up in CH_2Cl_2 (75 mL) and cooled in an ice bath. $[Cp^*Ru(NCCH_3)_3]PF_6$ (0.757 g, 1.50 mmol, 2.0 mol %) was added and the mixture was stirred overnight at room temperature. The mixture was diluted with Et_2O and filtered through a plug of Florisil with additional Et_2O. The filtrate was concentrated by rotary evaporation and the residue purified by Kugelrohr distillation (bp = 155-165 °C at 1 atm) to yield **8a** as a colorless oil (4.52 g, 31.8 mmol, 45%). IR (neat, diamond ATR): 2956, 2924, 2854, 1613, 1252, 1144, 1087, 1046, 864, 822, 779, 664 cm^{-1}; ^1H NMR ($CDCl_3$, 500 MHz): δ = 6.37 (tq, *J* = 4.0, 1.5 Hz, 1H), 3.91 (t, *J* = 5.5 Hz, 2 H), 2.22 (tdq, *J* = 5.5, 4.0, 1.5 Hz, 2H), 1.71 (dt, *J* = 1.5, 1.5 Hz, 3H), 0.18 (s, 6H); ^{13}C NMR ($CDCl_3$, 125 MHz): δ = 140.1, 134.9, 62.0, 30.7, 20.3, -2.0; HRMS (CI, NH_3): calcd for $C_7H_{18}NOSi$ (M+NH_4): 160.1158; found: 160.1161. *Anal.* Calcd for $C_7H_{14}OSi$: C, 59.09; H, 9.92. Found: C, 58.84; H, 9.73.

[2] Barbot, F.; Mesnard, D.; Miginiac, L. *Org. Prep. Proc. Int.*, **1978**, 10, 261-6.

2,2,3-Trimethyl-2,5,6,7-tetrahydro-1,2-oxasilepine (8b). The procedure for the preparation of **8a** was followed using 4-hexyn-1-ol (**6**)[3] (0.308 g, 3.14 mmol), 1,1,3,3-tetramethyldisilazane (1.42 mL, 1.07 g, 8.00 mmol), and [Cp*Ru(NCCH$_3$)$_3$]PF$_6$ (0.060 g, 0.119 mmol, 3.8 mol %) gave **8b** (0.4504 g, 2.88 mmol, 93%) as a colorless oil after distillation (bp = 78-92 °C at 100 mmHg). IR (neat, diamond ATR): 2926, 2871, 1619, 1250, 1136, 1107, 1095, 917, 841, 818, 783, 733, 655 cm^{-1}; ^{1}H NMR (CDCl$_3$, 300 MHz): δ = 6.27 (tq, *J* = 4.0, 2.0 Hz, 1H), 3.89 (t, *J* = 5.5 Hz, 2 H), 2.30-2.20 (m, 2H), 1.83 (quintet, *J* = 5.5 Hz, 2H) 1.74 (dt, *J* = 2.0, 2.0 Hz, 3H), 0.21 (s, 6H); ^{13}C NMR (CDCl$_3$, 125 MHz): δ = 141.7, 137.2, 64.3, 30.7, 28.5, 22.4, -1.3; HRMS (CI, NH$_3$): calcd for $C_8H_{20}NOSi$ (M+NH$_4$): 174.1314; found: 174.1307.

2,2,3,7,7-Pentamethyl-2,5,6,7-tetrahydro-1,2-oxasilepine (8c). The procedure for the preparation of **8a** was followed using 2-methyl-5-heptyn-2-ol (**7**) (1.88 g, 15.0 mmol), 1,1,3,3-tetramethyldisilazane (8.00 mL, 6.02 g, 45.0 mmol), and [Cp*Ru(NCCH$_3$)$_3$]PF$_6$ (0.151 g, 0.299 mmol, 2.0 mol %) gave **8c** (2.66 g, 14.4 mmol, 88%) as a slightly green oil after distillation (bp = 100-110 °C at 30 mmHg). IR (neat, diamond ATR): 2973, 2917, 1623, 1252, 1166, 1045, 907, 856, 827, 778, 678, 648 cm^{-1}; ^{1}H NMR (CDCl$_3$, 300 MHz): δ = 6.24 (tq, *J* = 4.0, 1.8 Hz, 1H), 2.30-2.20 (m, 2H), 1.80-1.70 (m, 2H) 1.60-1.50 (m, 3H), 1.25 (s, 6H), 0.18 (s, 6H); ^{13}C NMR (CDCl$_3$, 125 MHz): δ = 142.6, 135.9, 73.8, 42.4, 30.5, 26.7, 22.4, 0.8. *Anal.* Calcd for $C_{10}H_{20}OSi$: C, 65.15; H, 10.94. Found: C, 65.08; H, 11.15.

(*Z*)-4-(2,5-Dimethoxy-4-methylphenyl)pent-3-en-1-ol (10). The following were charged to a screw-cap culture tube: siloxane **8a** (0.101 g, 0.703 mmol, 1.0 equiv), THF (4 mL), 1-iodo-2,5-dimethoxy-4-methylbenzene (**9**) (0.293 g, 1.05 mmol, 1.5 equiv), and TBAF (1.40 mL 1 M in THF, 1.40 mmol) followed by Pd$_2$(dba)$_3$ (0.064 g, 0.070 mmol, 10 mol %). The tube was capped and after stirring 5 min, the tube was placed in a 50 °C oil bath for 18 h. The cooled mixture was filtered through a plug of silica gel, rinsing the plug with

---

[3] Arnold, H.; Overman, L. E.; Sharp, M. J.; Witschel, M. C. *Org. Synth. Coll. Vol. IX*, J. P. Freeman, Ed.; Wiley, 1998, 46.

Et_2O. The filtrate was concentrated by rotary evaporation and purified by flash chromatography (2:1 hexanes:EtOAc) to provide **10** as a pale yellow oil (0.152 g, 0.643 mmol, 92%). R_f = 0.30 (2:1 hexanes:EtOAc); IR (neat, diamond ATR): 3356 (br), 2934, 1502, 1465, 1208, 1041, 865 cm^{-1}; ^1H NMR ($CDCl_3$, 300 MHz): δ = 6.73 (s, 1H), 6.53 (s, 1H), 5.51 (t, *J* = 7.5 Hz, 1H), 3.78 (s, 3H), 3.77 (s, 3H), 3.56 (br t, *J* = 6.5 Hz, 2H), 2.23 (s, 3H), 2.10 (app q, *J* = 6.5 Hz, 2H), 2.00 (s, 3H); ^{13}C NMR ($CDCl_3$, 75 MHz): δ = 151.8, 149.3, 137.6, 128.4, 125.9, 124.3, 114.4, 111.9, 62.0, 56.2, 56.0, 32.8, 25.2, 16.3; HRMS (ESI): calcd for $C_{14}H_{20}NaO_3$ (M+Na): 259.1310; found: 259.1311. *Anal.* Calcd for $C_{14}H_{20}O_3$: C, 71.16; H, 8.53. Found: C, 71.44; H, 8.72.

(Z)-5-(2,5-Dimethoxy-4-methylphenyl)hex-4-en-1-ol (11). The procedure used for the preparation of **10** was followed. Siloxane **8b** (0.166 g, 1.00 mmol), THF (4 mL), iodide **9** (0.417 g, 1.50 mmol, 1.5 equiv), TBAF (2.0 mL, 1M in THF, 2.0 mmol) and $Pd_2(dba)_3$ (0.0229 g, 0.0250 mmol, 2.5 mol %) gave **11** (0.129 g, 0.515 mmol, 52%) as a pale yellow oil after flash chromatography (3:1 hexanes:EtOAc). R_f = 0.20 (3:1 hexanes:EtOAc); IR (neat, diamond ATR): 3363 (br), 2933, 1502, 1465, 1397,1206, 1045, 863, 801 cm^{-1}; ^1H NMR ($CDCl_3$, 500 MHz): δ = 6.74 (s, 1H), 6.52 (s, 1H), 5.50 (tq, *J* = 7.3, 1.5 Hz, 1H), 3.78 (s, 3H), 3.77 (s, 3H), 3.57 (br t, *J* = 6.5 Hz, 2H), 2.23 (s, 3H), 1.98 (q, *J* = 1.5 Hz, 3H), 1.93 (app qq, *J* = 7.3, 1.5 Hz, 2H), 1.84 (br s, 1H, OH), 1.59 (quintet, *J* = 6.5 Hz, 2H); ^{13}C NMR ($CDCl_3$, 125 MHz): δ = 151.7, 149.6, 134.6, 128.8, 127.7, 125.8, 114.6, 112.1, 62.4, 56.3, 56.0, 32.3, 25.8, 24.9, 16.3; HRMS (ESI): calcd for $C_{15}H_{22}NaO_3$ (M+Na): 273.1467; found: 273.1467. *Anal.* Calcd for $C_{15}H_{22}O_3$: C, 71.97; H, 8.86. Found: C, 72.23; H, 8.64.

(*Z*)-6-(2-Bromophenyl)-2-methylhept-5-en-2-ol (13). The procedure used for the preparation of **10** was followed. Siloxane **8c** (0.563 g, 3.05 mmol), THF (8 mL), iodide **12** (0.58 mL, 1.28 g, 4.52 mmol, 1.5 equiv), TBAF (6.00 mL, 1 M in THF, 6.00 mmol) and $Pd_2(dba)_3$ (0.077 g, 0.084 mmol, 2.8 mol %) gave **13** (0.750 g, 2.65 mmol, 87%) as a pale yellow oil after flash chromatography (4:1 hexanes:EtOAc). R_f = 0.25 (4:1 hexanes:EtOAc); IR (neat, diamond ATR): 3368, 3052, 2968, 2930, 2850, 1590, 1560, 1469, 1429, 1373, 1144, 1022, 905, 754, 729, 654 cm^{-1}; ^1H NMR ($CDCl_3$, 300 MHz): δ = 7.57 (dd, *J* = 7.5, 1.0 Hz, 1H), 7.26 (td, *J* = 7.5, 1.0 Hz, 1H), 7.20-7.10 (m, 2H), 5.55 (tq, *J* = 7.5, 1.5 Hz, 1H), 1.97 (q, *J* = 1.5 Hz, 3H), 1.90-1.70 (m, 2H),

1.50-1.40 (m, 2H), 1.09 (s, 6H); ^{13}C NMR ($CDCl_3$, 125 MHz): δ = 143.0, 136.4, 132.7, 129.9, 128.6, 128.2, 127.4, 122.4, 70.9, 43.1, 29.7, 29.0, 24.4; *Anal.* Calcd for $C_{14}H_{19}BrO$: C, 59.37; H, 6.76. Found: C, 58.95; H, 6.73.

(*Z*)-6-(2-Bromo-4-methylphenyl)-2-methylhept-5-en-2-ol (15). The procedure used for the preparation of **10** was followed. Siloxane **8c** (1.05 g, 5.70 mmol), THF (12 mL), iodide **14** (2.53 g, 8.54 mmol, 1.5 equiv), TBAF (11.4 mL, 1 M in THF, 11.4 mmol) and $Pd_2(dba)_3$ (0.145 g, 0.158 mmol, 2.8 mol %) gave **15** (1.59 g, 5.35 mmol, 94%) as a pale yellow oil after flash chromatography (3:1 hexanes:EtOAc). R_f = 0.45 (3:1 hexanes:EtOAc); IR (neat, diamond ATR): 3379, 2969, 2925, 1604, 1489, 1375, 1208, 1149, 1035, 908, 853, 821, 734 cm^{-1}; ^{1}H NMR ($CDCl_3$, 500 MHz): δ = 7.40 (d, *J* = 1.0 Hz, 1H), 7.08 (dd, *J* = 7.8, 1.0 Hz, 1H), 6.97 (d, *J* = 7.8 Hz, 1H), 5.51 (tq, *J* = 7.3, 1.5 Hz, 1H), 2.32 (s, 3H), 1.95 (q, *J* = 1.5 Hz, 3H), 1.80-1.70 (m, 2H), 1.50-1.40 (m, 2H), 1.10 (s, 6H); ^{13}C NMR ($CDCl_3$, 125 MHz): δ = 139.9, 138.2, 136.3, 133.1, 129.6, 128.7, 128.2, 122.1, 70.9, 43.2, 29.0, 24.5, 24.3, 20.7. *Anal.* Calcd for $C_{15}H_{21}BrO$: C, 60.61; H, 7.12. Found: C, 60.25; H, 7.01.

1-Bromo-4-chloro-2-iodo-5-methylbenzene (16). A solution of 2-bromo-5-chloro-4-methylaniline[4] (7.70 g, 35.0 mmol) in water (145 mL) and conc. HCl (143 mL) was cooled in an ice bath. $NaNO_2$ (2.67 g, 38.7 mmol) dissolved in water (5 mL) was added to the solution keeping the internal temperature below 10 °C. The mixture was stirred for 30 min at ice bath temperature. KI (6.41 g, 38.7 mmol) was dissolved in water (5 mL) and added to the mixture. The resulting dark brown solution was stirred overnight as it slowly warmed to room temperature. The suspension was extracted with CH_2Cl_2 (150 mL) and the organic layer was washed successively with 10% NaOH (150 mL), 1 M $Na_2S_2O_3$ (150 mL), 10% HCl (150 mL), sat $NaHCO_3$ (150 mL) and brine (150 mL). The organic layer was dried over $MgSO_4$, filtered and concentrated via rotary evaporation. Recrystallization of the resulting material from EtOH gave **16** as a white solid (5.98 g, 18.1 mmol, 51%). Mp = 80.0 – 80.5 °C; IR (neat, diamond ATR): 3073, 2919, 1571, 874, 860, 759, 703 cm^{-1}; ^{1}H NMR ($CDCl_3$, 500

[4] Bavetsias, V.; Skelton, L. A.; Yafai, F.; Mitchell, F.; Wilson, S. C.; Allan, B.; Jackman, A. L. *J. Med. Chem.*, **2002**, 45, 3692-3702.

MHz): δ = 7.79 (s, 1H), 7.48 (s, 1H), 2.30 (s, 3H); ^{13}C NMR ($CDCl_3$, 75 MHz): δ = 139.4, 137.9, 134.0, 133.8, 127.5, 97.4, 19.6; *Anal.* Calcd for C_7H_7ClBrI: C, 25.37; H, 1.52. Found: C, 25.12; H, 1.33.

(*Z*)-6-(2-Bromo-5-chloro-4-methylphenyl)-2-methylhept-5-en-2-ol (17). The procedure used for the preparation of **10** was followed. Siloxane **8c** (0.336 g, 2.00 mmol), THF (4 mL), iodide **16** (0.994 g, 3.00 mmol, 1.5 equiv), TBAF (4.00 mL, 1 M in THF, 4.00 mmol) and $Pd_2(dba)_3$ (0.046 g, 0.050 mmol, 2.5 mol %) gave **17** (0.532 g, 1.60 mmol, 80%) as a pale yellow oil after flash chromatography (6:1 hexanes:EtOAc). R_f = 0.35 (6:1 hexanes:EtOAc); IR (neat, diamond ATR): 3377, 2968, 2926, 1475, 1443, 1146, 1062, 885 cm^{-1}; ^{1}H NMR ($CDCl_3$, 500 MHz): δ = 7.44 (s, 1H), 7.07 (s, 1H), 5.52 (tq, *J* – 7.2, 2.5 Hz, 1H), 2.37 (s, 3H), 1.93 (d, *J* = 2.5 Hz, 3H), 1.80-1.70 (m, 2H), 1.26 (t, *J* = 7.3 Hz, 2H), 1.12 (s, 6H); ^{13}C NMR ($CDCl_3$, 75 MHz): δ = 141.8, 136.1, 135.0, 134.5, 133.3, 129.8, 129.3, 120.0, 70.9, 43.0, 29.0, 24.4, 24.2, 19.5.

1-(4-Bromo-5-iodo-2-methylphenyl)ethan-1-one (18). A solution of $CaCO_3$ (4.688 g, 4.700 mmol) in water (12 mL) was added to a solution of 4-amino-2-methylacetophenone[5] (4.044 g, 30.00 mmol) in MeOH (20 mL). This was followed by dropwise addition of a solution of ICl (5.02 g, 31.8 mmol) in MeOH (20 mL). The mixture was stirred at room temperature of 18 h, then diluted with Et_2O (100 mL) and quenched with water (50 mL). The aqueous layer was extracted with ether (100 mL) and the combined organic layers were dried over Na_2SO_4. Filtration and concentration by rotary evaporation gave a crude product that was recrystallized from EtOH to give 4-amino-5-iodo-2-methylacetophenone (4.917 g, 17.10 mmol, 57%) as a yellow solid that was used in the next reaction. Mp = 118-120 °C; IR (neat, diamond ATR): 3196, 2966, 1650, 1625, 1588, 1445, 1253 cm^{-1}; ^{1}H NMR ($CDCl_3$, 500 MHz): δ = 8.07 (s, 1H), 6.54 (s, 1H), 4.46 (s, 2H, NH_2), 2.50 (s, 3H), 2.47 (s, 3H); ^{13}C NMR ($CDCl_3$, 75 MHz): δ = 197.5, 149.8, 142.3, 138.7, 129.0, 116.9, 78.2, 28.7, 22.4.

A solution of $NaNO_2$ (1.239 g, 18.00 mmol) in water (16 mL) was added dropwise over 15 min to a mixture of 4-amino-5-iodo-2-methylacetophenone (4.133 g, 15.00 mmol) and HBr (47% w/w, 36 mL) that had been cooled to -10 °C. The mixture was stirred for 10 min before warming to 0 °C and stirring 2 h. This solution was then

[5] Royer, R.; Eckert, B. *J. Org. Chem.*, **1952**, 17, 1463-1465.

added dropwise over 30 min to a vigorously stirred mixture of CuBr (2.601 g, 18.00 mmol) and HBr (47%, 20 mL) that was heated to 60 °C. The mixture was stirred an additional 30 min at 80 °C before cooling to room temperature. The mixture was diluted with water (200 mL) and extracted with EtOAc (200 mL). The organic layer was washed with 1 M HCl (150 mL), sat $NaHCO_3$, and brine (125 mL). The organic layer was dried over $MgSO_4$, filtered and concentrated by rotary evaporation. The crude product was purified by flash chromatography (3:1 hexanes:EtOAc) to give iodide **18** (1.443 g, 4.230 mmol, 28%) as an oil. R_f = 0. 50 (3:1 hexanes:EtOAc); IR (neat, diamond ATR): 2974, 2923, 1674, 1570, 1526, 1235, 1125, 955, 884, 863 cm^{-1}; ^1H NMR ($CDCl_3$, 500 MHz): δ = 8.09 (s, 1H), 7.53 (s, 1H), 2.55 (s, 3H), 2.44 (s, 3H); ^{13}C NMR ($CDCl_3$, 75 MHz): δ = 199.0, 140.5, 140.0, 137.6, 135.8, 133.3, 96.9, 29.3, 21.0.

(*Z*)-1-(4-Bromo-5-(6-hydroxy-6-methylhept-2-en-2-yl)-2-methylphenyl)ethan-1-one (19). The procedure used for the preparation of **10** was followed. Siloxane **8c** (0.226 g, 1.33 mmol), THF (4 mL), iodide **18** (0.646 g, 2.00 mmol, 1.5 equiv), TBAF (2.60 mL, 1 M in THF, 2.60 mmol) and $Pd_2(dba)_3$ (0.033 g, 0.036 mmol, 2.7 mol %) gave **19** (0.260 g, 0.798 mmol, 60%) as a pale yellow oil after flash chromatography (3:1 hexanes:EtOAc). R_f = 0.20 (3:1 hexanes:EtOAc); IR (neat, diamond ATR): 3331, 2964, 2927, 2874, 1684, 1594, 1533, 1435, 1377, 1356, 1272, 1245, 1152, 1067, 958, 887 cm^{-1}; ^1H NMR ($CDCl_3$, 300 MHz): δ = 7.48 (s, 1H), 7.40 (s, 1H), 5.52 (br t, *J* = 6.5 Hz, 1H), 2.56 (s, 3H), 2.47 (s, 3H), 1.93 (br s, 3H), 1.80-1.70 (m, 2H), 1.26 (t, *J* = 6.5 Hz, 2H), 1.12 (s, 6H); ^{13}C NMR ($CDCl_3$, MHz): δ = 200.6, 140.6, 138.6, 136.7, 136.0, 135.3, 130.6, 129.6, 126.1, 70.8, 43.1, 29.5, 24.2, 21.1, 19.8, 13.7.

(*Z*)-6-(2-Methoxyphenyl)-2-methylhept-5-en-2-ol (21). The procedure used for the preparation of **10** was followed. Siloxane **8c** (1.127 g, 6.110 mmol), THF (12 mL), iodide **20** (2.15 g, 9.10 mmol, 1.5 equiv), TBAF (12.2 mL, 1 M in THF, 12.2 mmol) and $Pd_2(dba)_3$ (0.139 g, 0.152 mmol, 2.5 mol %) gave **21** (0.685 g, 2.92 mmol, 48%) as a pale yellow oil after flash chromatography (3:1 hexanes:EtOAc). R_f = 0.35 (3:1 hexanes:EtOAc); IR (neat, diamond ATR): 3381, 2967, 1598, 1491, 1465, 1433, 1375, 1245, 1150, 1046, 905, 801, 753 cm^{-1}; ^1H NMR ($CDCl_3$, 500 MHz): δ = 7.24 (td, *J* = 7.5, 2.0 Hz, 1H), 7.02 (dd, *J* = 7.5, 2.0 Hz, 1H),

6.94 (td, J = 7.5, 1.0 Hz, 1H), 6.90 (br d, J = 7.5 Hz, 1H), 5.53 (br t, J = 6.5 Hz, 1H), 3.80 (s, 3H), 1.97 (br s, 3H), 1.90 (app q, J = 6.5 Hz, 2H), 1.50-1.40, m, 2H), 1.08 (s, 6H); ^{13}C NMR ($CDCl_3$, 125 MHz): δ = 155.6, 142.4, 133.6, 129.2, 127.8, 127.4, 120.0, 110.2, 70.5, 54.8, 44.4, 42.6, 29.0, 28.4, 24.1; *Anal.* Calcd for $C_{15}H_{22}O_2$: C, 76.88; H, 9.46. Found: C, 76.52; H, 9.64.

(*Z*)-6-(2,6-Dimethylpyridin-3-yl)-2-methylhept-5-en-2-ol (23). The procedure used for the preparation of **10** was followed. Siloxane **8c** (0.0931 g, 0.504 mmol), THF (2 mL), iodide **22** (0.0950 g, 0.408 mmol), TBAF (0.92 mL, 1M in THF, 0.92 mmol) and $Pd_2(dba)_3$ (0.0102 g, 0.0111 mmol, 2.7 mol %) gave **23** (0.0544 g, 0.237 mmol, 58%) as a pale yellow oil after flash chromatography (EtOAc). R_f = 0.35 (EtOAc); IR (neat, diamond ATR): 3350, 2967, 1926, 1591, 1564, 1466, 1432, 1148, 1026, 906, 828, 731 cm^{-1}; ^{1}H NMR ($CDCl_3$, 300 MHz): δ = 7.20 (d, J = 7.9 Hz, 1H), 6.96 (d, J = 7.9 Hz, 1H), 5.52 (tq, J = 7.5, 1.3 Hz, 1H), 2.51 (s, 3H), 2.41 (S, 3H), 1.91 (q, J = 1.3 Hz), 1.80-1.70 (m, 2H), 1.50-1.40 (m, 2H), 1.09 (s, 6H); ^{13}C NMR ($CDCl_3$, 75 MHz): δ = 155.8, 154.4, 136.6, 134.4, 133.5, 128.8, 120.5, 70.7, 43.2, 29.0, 24.9, 24.1, 23.9, 21.8; HRMS: calcd for $C_{15}H_{23}NONa$ (M+Na): 256.1677; found: 256.1685.

(*Z*)-2,2,6,9-Tetramethyl-3,4-dihydro-2*H*-benzo[*b*]oxocine (24) and (*Z*)-2-methyl-6-(*p*-tolyl)hept-5-en-2-ol (25). A small screw-cap vial was charged with bromide **15** (0.119 g, 0.400 mmol), $Pd_2(dba)_3$ (0.0366 g. 0.0399 mmol, 10 mol %), CTC Q-Phos (0.0285 g, 0.0401 mmol, 10 mol %), and NaO*t*-Bu (0.0460 g, 0.479 mmol). Toluene was added (2 mL), the vial was flushed with argon, capped, and heated in an 80 °C oil bath for 24 h. The mixture was filtered through a silica plug and concentrated. Flash chromatography (19:1 hexanes:EtOAc gradient to 3:1 hexanes:EtOAc) gave **24** (0.0082 g, 0.0379 mmol, 10%) and **25** (0.0108 g, 0.0495 mmol, 12%) as pale yellow oils.

(*Z*)-2,2,6,9-tetramethyl-3,4-dihydro-2*H*-benzo[b]oxocine (24): R_f = 0.40 (19:1 hexanes:EtOAc); IR (neat, diamond ATR): 3024, 2972, 2923, 2856, 1610, 1560, 1500, 1382, 1365, 1283, 1237, 1217, 1170, 1139, 1126, 961, 822 cm^{-1}; ^{1}H NMR ($CDCl_3$, 500 MHz): δ = 7.13 (d, J = 7.8 Hz, 1H), 6.91 (dd, J = 7.8, 1.0 Hz, 1H), 6.80 (d, J = 1.0 Hz), 1H), 5.70 (br t, J = 6.5 Hz, 1H), 2.31 (s, 3H), 2.20-2.10 (m, 2H), 1.98 (s, 3H), 1.50-1.40 (m,

2H), 1.34 (s, 6H); ^{13}C NMR ($CDCl_3$, 125 MHz): δ = 151.7, 136.7, 134.4, 130.4, 128.1, 126.64, 126.59, 124.5, 77.5, 35.4, 27.9, 26.2, 24.7, 21.1; MS (EI) *m/z* (rel int) = 216(100), 161(15), 159(46), 145(14); HRMS: calcd for $C_{15}H_{20}O$: 216.1514; found: 216.1509.

(*Z*)-2-Methyl-6-(*p*-tolyl)hept-5-en-2-ol (25): R_f = 0.45 (3:1 hexanes:EtOAc); ^1H NMR ($CDCl_3$, 500 MHz): δ = 7.14 (d, *J* = 7.5 Hz, 2H), 7.07 (d, *J* = 7.5 Hz, 2H), 5.45 (tq, *J* = 7.5, 1.5 Hz, 1H), 2.35 (s, 3H), 2.15-2.00 (m, 2H), 2.00 (q, *J* = 1.5 Hz, 3H), 1.65-1.45 (m, 2H), 1.13 (s, 6H); ^{13}C NMR ($CDCl_3$, 125 MHz): δ = 138.9, 136.3, 136.1, 128.8, 127.7, 127.2, 71.0, 44.0, 29.1, 25.6, 24.3, 21.1; HRMS: calcd for $C_{15}H_{20}$ (M-H_2O): 200.1566; found: 200.1547.

2,2,6,9-Tetramethyl-3,4,5,6-tetrahydro-2*H*-benzo[*b*]oxocine (26). Ether **24** (0.0286 g, 0.132 mmol) was dissolved in EtOH (1.5 mL) and Pd on carbon (10%, 0.032 g, 0.030 mmol) was suspended in the solution. The mixture was flushed with argon before flushing the flask with hydrogen. A hydrogen balloon was affixed to the flask and the mixture was stirred overnight. The flask was flushed with argon, the mixture was filtered through a plug of silica gel, eluting with 19:1 hexanes:EtOAc. The filtrate was concentrated by rotary evaporation to give **26** (0.0128 g, 0.0587 mmol, 44%) as a pale yellow oil. ^1H NMR ($CDCl_3$, 500 MHz): 7.10 (d, *J* = 7.8 Hz, 1H), 6.93 (d, *J* = 7.8 Hz, 1H), 6.75 (br s, 1H), 3.25-3.15 (br m, 1H), 2.29 (s, 3H), 1.80-1.70 (m, 1H), 1.70-1.45 (m, 3H), 1.45 (s, 3H), 1.45-1.30 (m, 2H), 1.35 (s, 3H), 1.25 (d, *J* = 7.0 Hz, 3H).

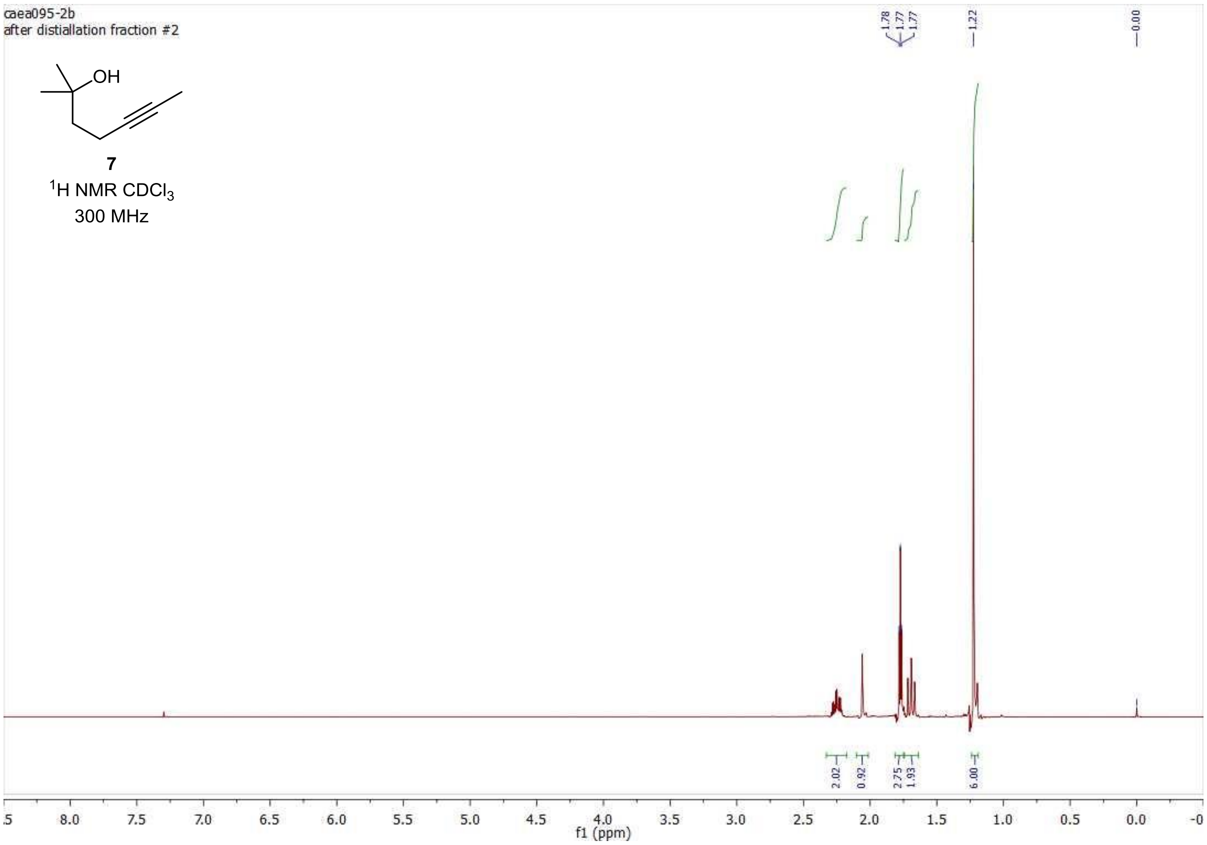
caea095-2b
after distiallation fraction #2
OH
7
^{1}H NMR $CDCl_3$
300 MHz
1.78
1.77
1.77
1.22
0.00
2.02
0.92
2.75
1.93
6.00
f1 (ppm)

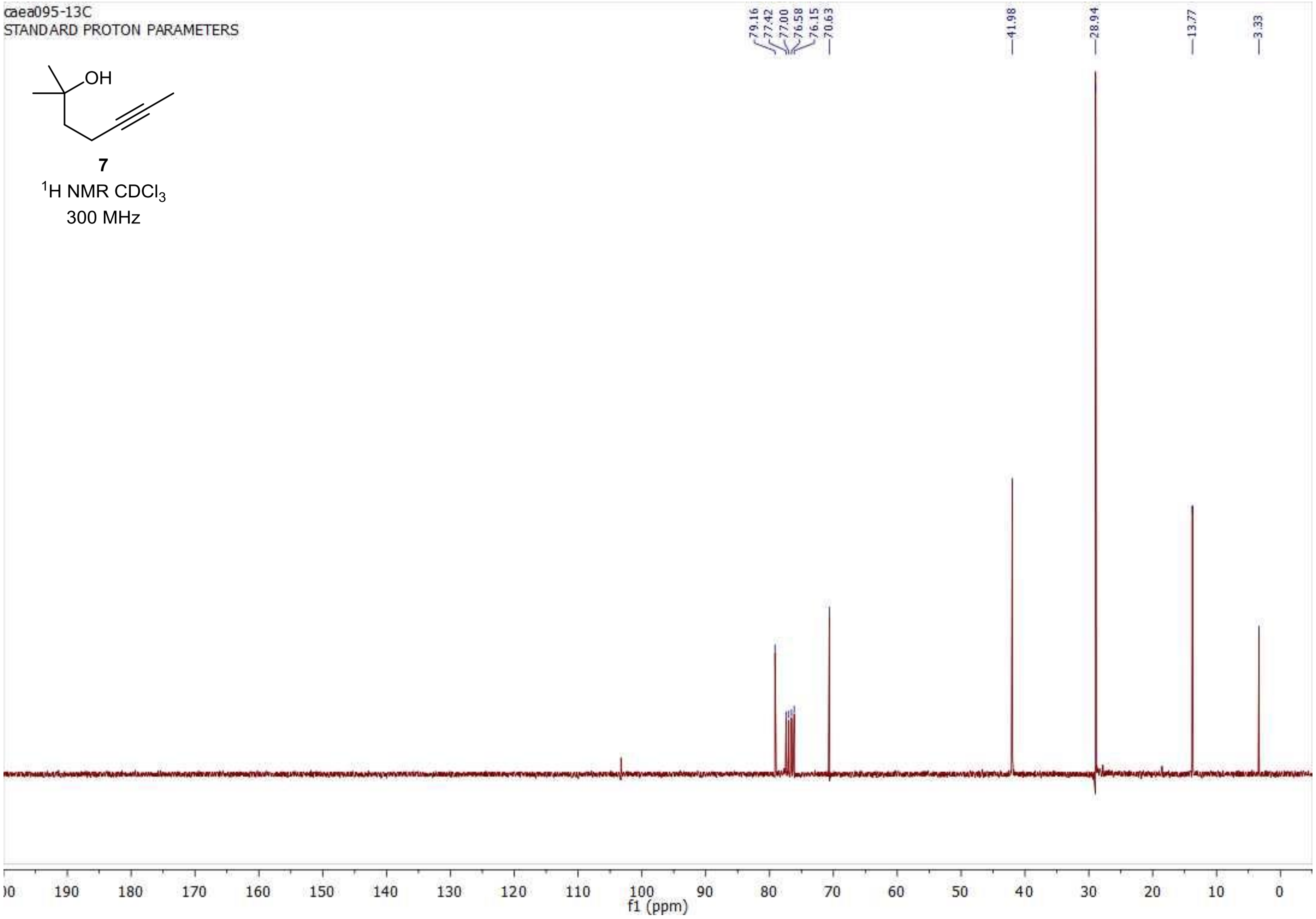
caea095-13C
STANDARD PROTON PARAMETERS
OH
7
^{1}H NMR $CDCl_3$
300 MHz
79.16
77.42
77.00
76.58
76.15
70.63
41.98
28.94
13.77
3.33
f1 (ppm)

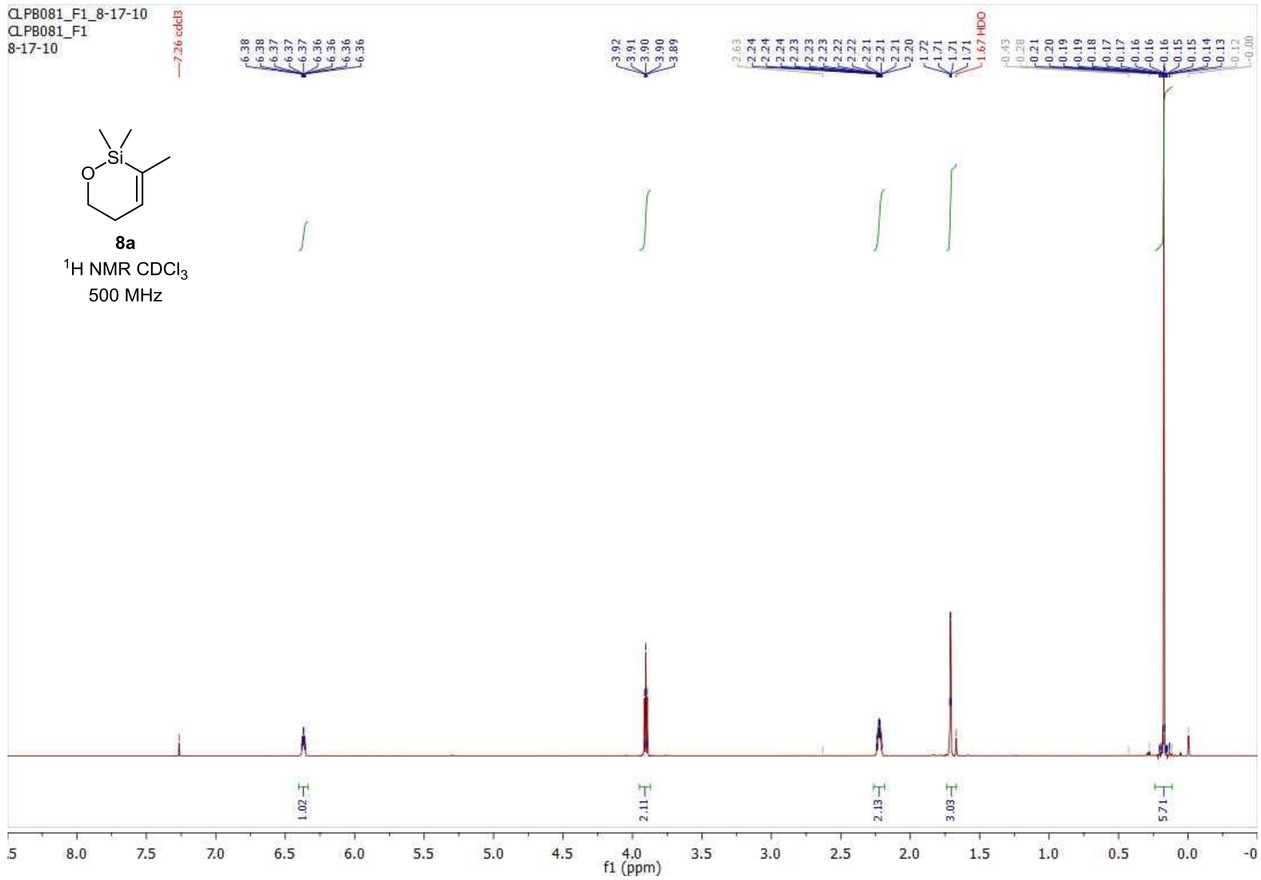
CLPB081_F1_8-17-10
CLPB081_F1
8-17-10
7.26 cdcl3
6.38
6.38
6.37
6.37
6.37
6.36
6.36
6.36
6.36
3.92
3.91
3.90
3.90
3.89
2.24
2.24
2.24
2.23
2.23
2.23
2.22
2.22
2.21
2.21
2.21
2.20
1.72
1.71
1.71
1.71
1.67 HDO
0.21
0.20
0.19
0.19
0.18
0.17
0.17
0.16
0.16
0.16
0.15
0.15
0.14
0.13
0.13
O
Si
8a
^{1}H NMR CDCl$_3$
500 MHz
1.02
2.11
2.13
3.03
5.71
8.0
7.5
7.0
6.5
6.0
5.5
5.0
4.5
4.0
3.5
3.0
2.5
2.0
1.5
1.0
0.5
0.0
f1 (ppm)

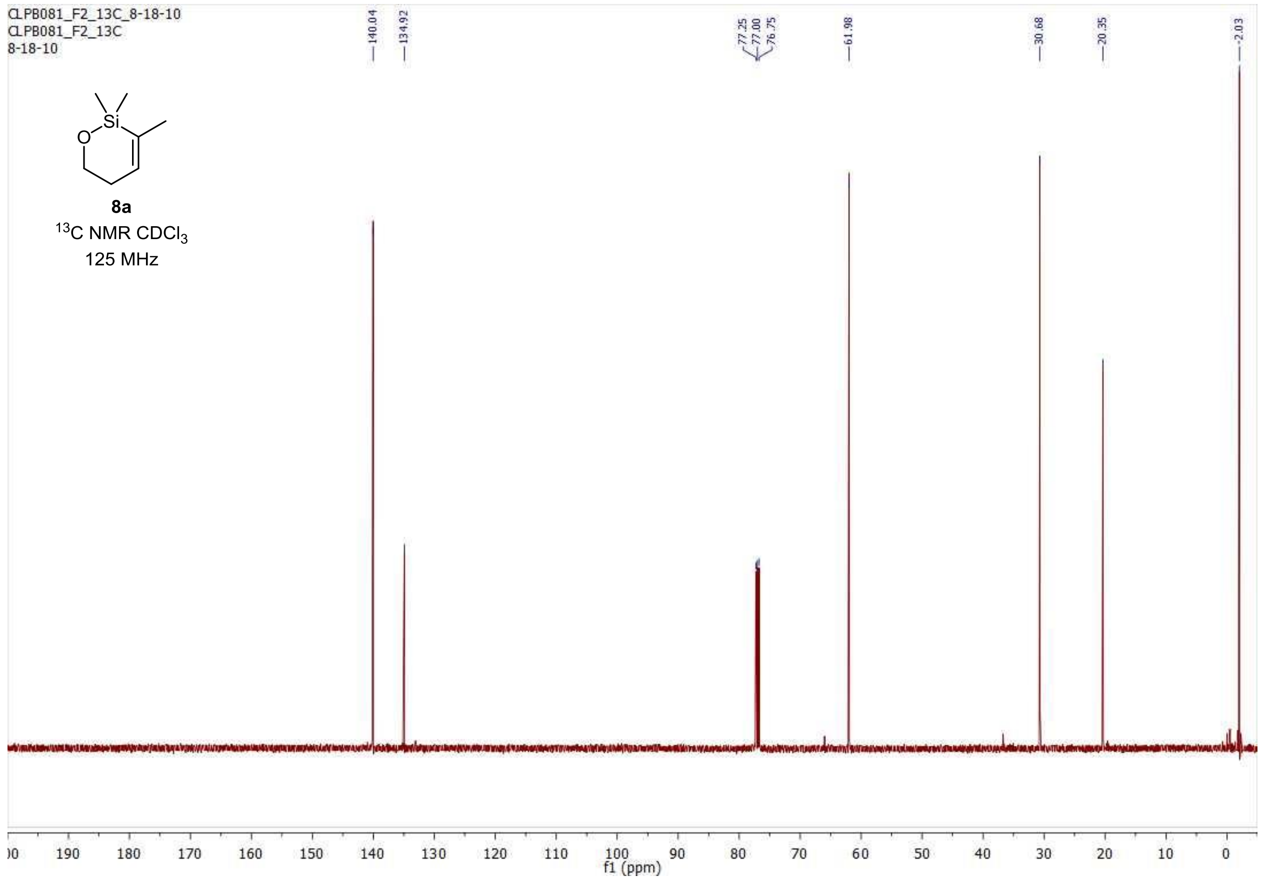
CLPB081_F2_13C_8-18-10
CLPB081_F2_13C
8-18-10
140.04
134.92
77.25
77.00
76.75
61.98
30.68
20.35
-2.03
O
Si
8a
^{13}C NMR CDCl$_3$
125 MHz
190
180
170
160
150
140
130
120
110
100
90
80
70
60
50
40
30
20
10
0
f1 (ppm)

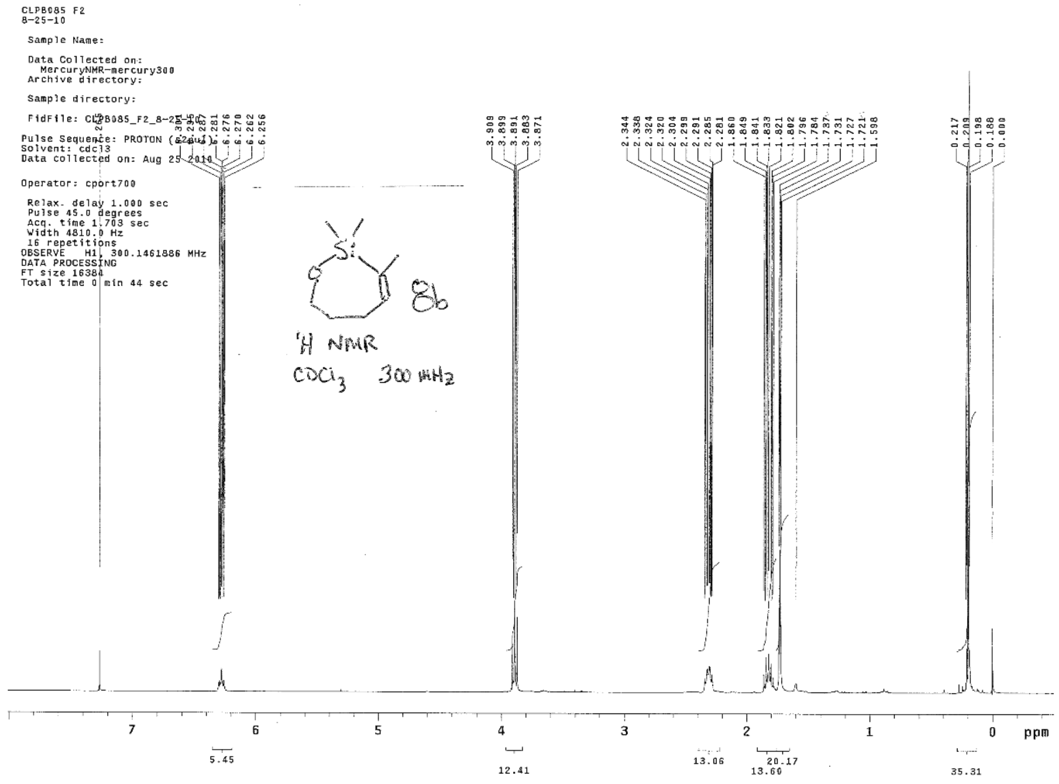
CLPB085 F2
8-25-10
Sample Name:
Data Collected on:
MercuryNMR-mercury300
Archive directory:
Sample directory:
Pulse Sequence: PROTON (s2pul)
Solvent: cdcl3
Operator: cport700
Relax. delay 1.000 sec
Pulse 45.0 degrees
Width 4810.0 Hz
16 repetitions
OBSERVE H1, 300.1461886 MHz
DATA PROCESSING
FT size 16384
Total time 0 min 44 sec
8b
¹H NMR
CDCl3 300 MHz
ppm
5.45
12.41
13.06
20.17
13.60
35.31

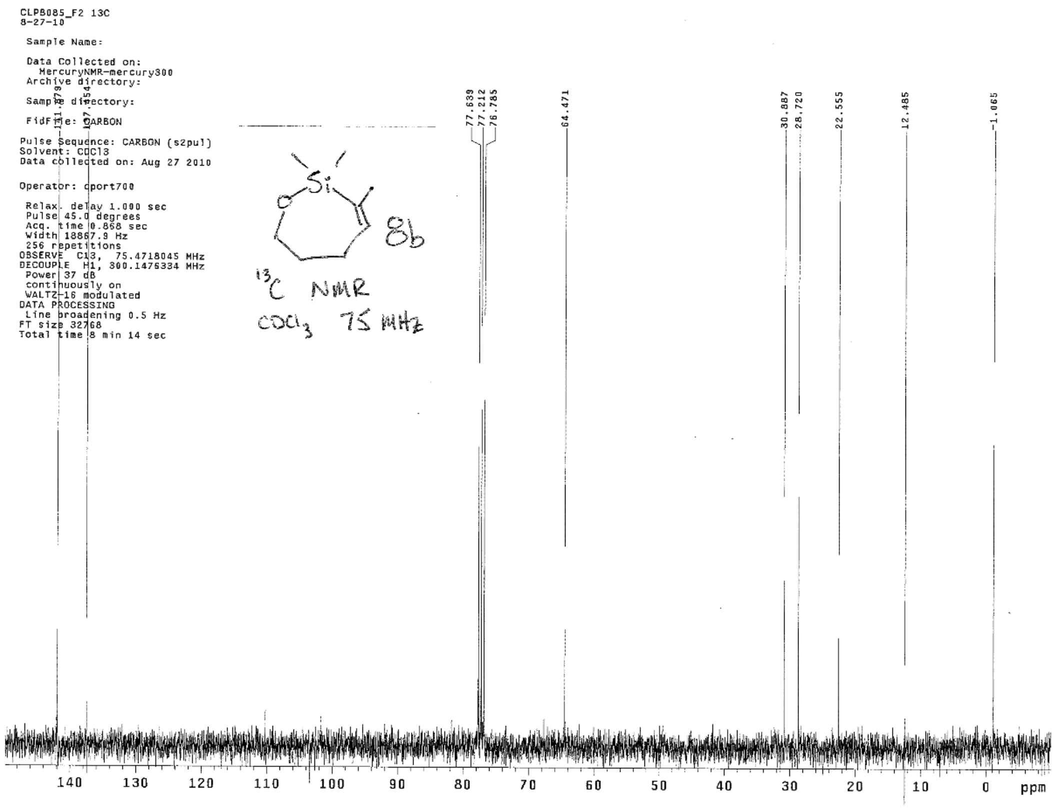
CLPB085_F2 13C
8-27-10
Sample Name:
Data Collected on:
MercuryNMR-mercury300
Archive directory:
Sample directory:
Pulse Sequence: CARBON (s2pul)
Solvent: CDCl3
Data collected on: Aug 27 2010
Operator: cport700
Relax. delay 1.000 sec
Pulse 45.0 degrees
Acq. time 0.868 sec
256 repetitions
OBSERVE C13, 75.4718045 MHz
DECOUPLE H1, 300.1476334 MHz
Power 37 dB
continuously on
WALTZ-16 modulated
DATA PROCESSING
Line broadening 0.5 Hz
FT size 32768
Total time 8 min 14 sec
77.639
77.212
76.785
64.471
30.887
28.720
22.555
12.485
-1.065
8b
¹³C NMR
CDCl3 75 MHz
ppm

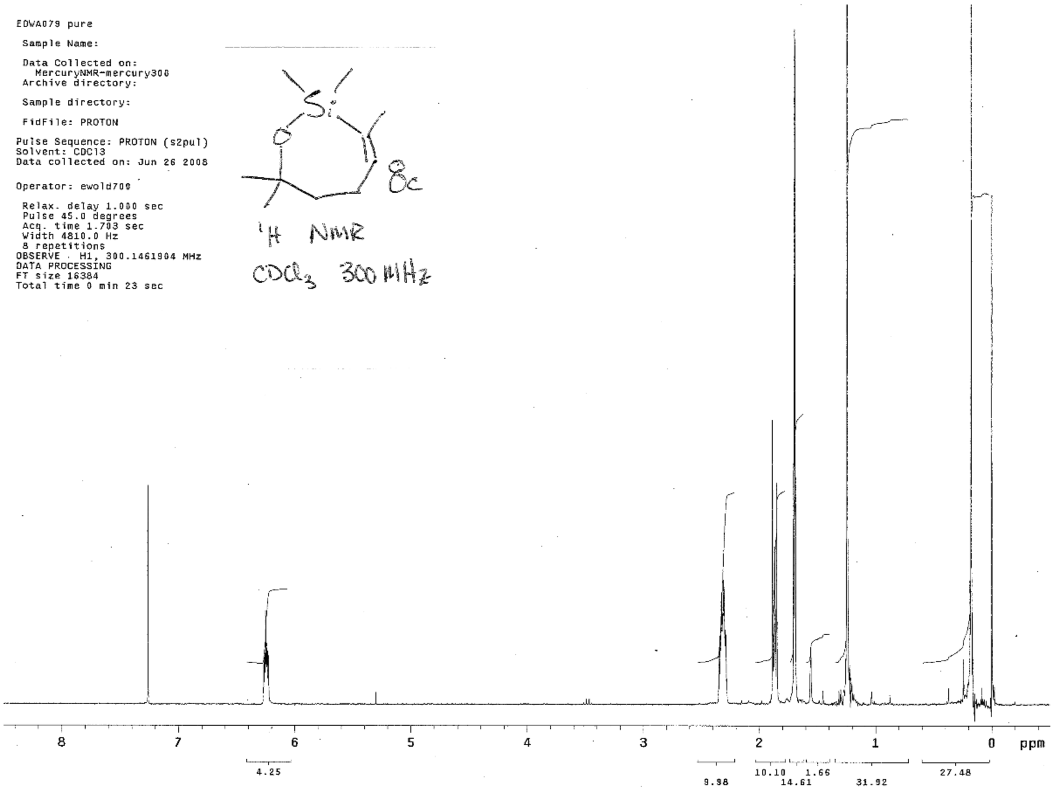
EDWA079 pure
Sample Name:
Data Collected on:
MercuryNMR-mercury300
Archive directory:
Sample directory:
FidFile: PROTON
Pulse Sequence: PROTON (s2pul)
Solvent: CDCl3
Data collected on: Jun 26 2008
Operator: ewold700
Relax. delay 1.000 sec
Pulse 45.0 degrees
Acq. time 1.703 sec
Width 4810.0 Hz
8 repetitions
OBSERVE H1, 300.1461904 MHz
DATA PROCESSING
FT size 16384
Total time 0 min 23 sec
Si
O
8c
1H NMR
CDCl3 300 MHz
8
7
6
5
4
3
2
1
0
ppm
4.25
9.98
10.10
14.61
1.66
31.92
27.48

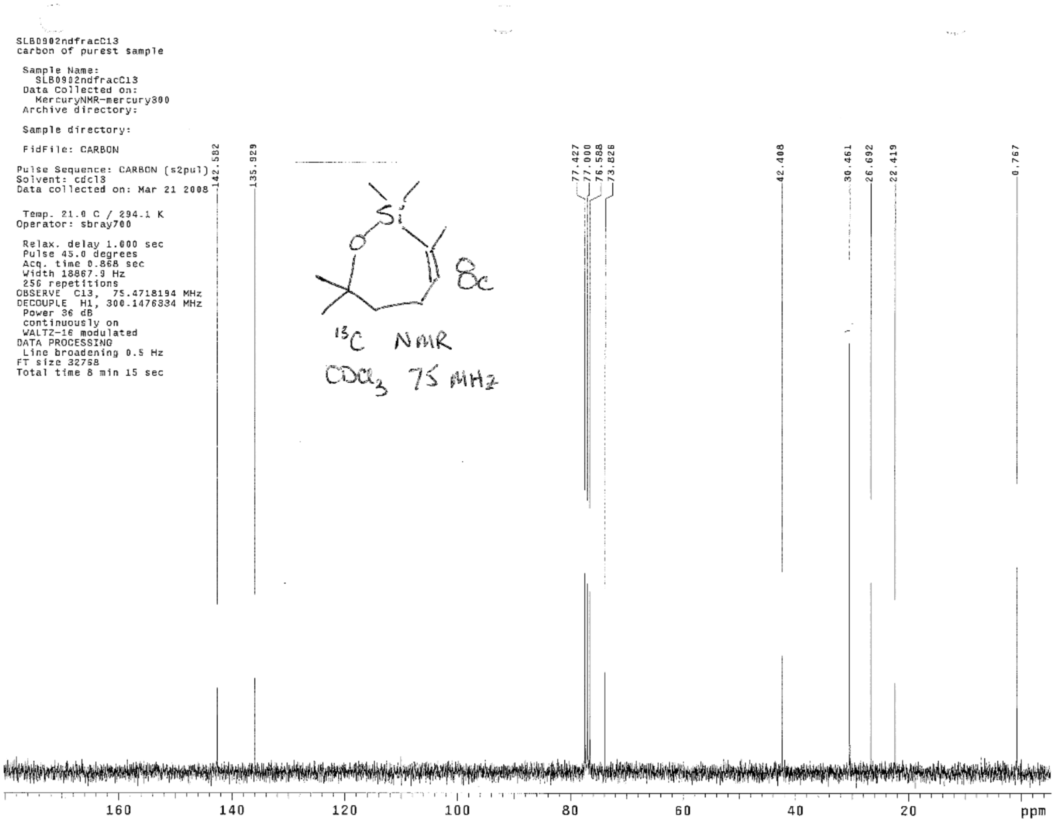
SLB0902ndfracC13
carbon of purest sample
Sample Name:
SLB0902ndfracC13
Data Collected on:
MercuryNMR-mercury300
Archive directory:
Sample directory:
FidFile: CARBON
Pulse Sequence: CARBON (s2pul)
Solvent: cdcl3
Data collected on: Mar 21 2008
Temp. 21.0 C / 294.1 K
Operator: sbray700
Relax. delay 1.000 sec
Pulse 45.0 degrees
Acq. time 0.868 sec
Width 18867.9 Hz
256 repetitions
OBSERVE C13, 75.4718194 MHz
DECOUPLE H1, 300.1476334 MHz
Power 36 dB
continuously on
WALTZ-16 modulated
DATA PROCESSING
Line broadening 0.5 Hz
FT size 32768
Total time 8 min 15 sec
142.582
135.929
77.427
77.000
76.588
73.826
42.408
30.461
26.692
22.419
0.767
Si
O
8c
13C NMR
CDCl3 75 MHz
160
140
120
100
80
60
40
20
ppm

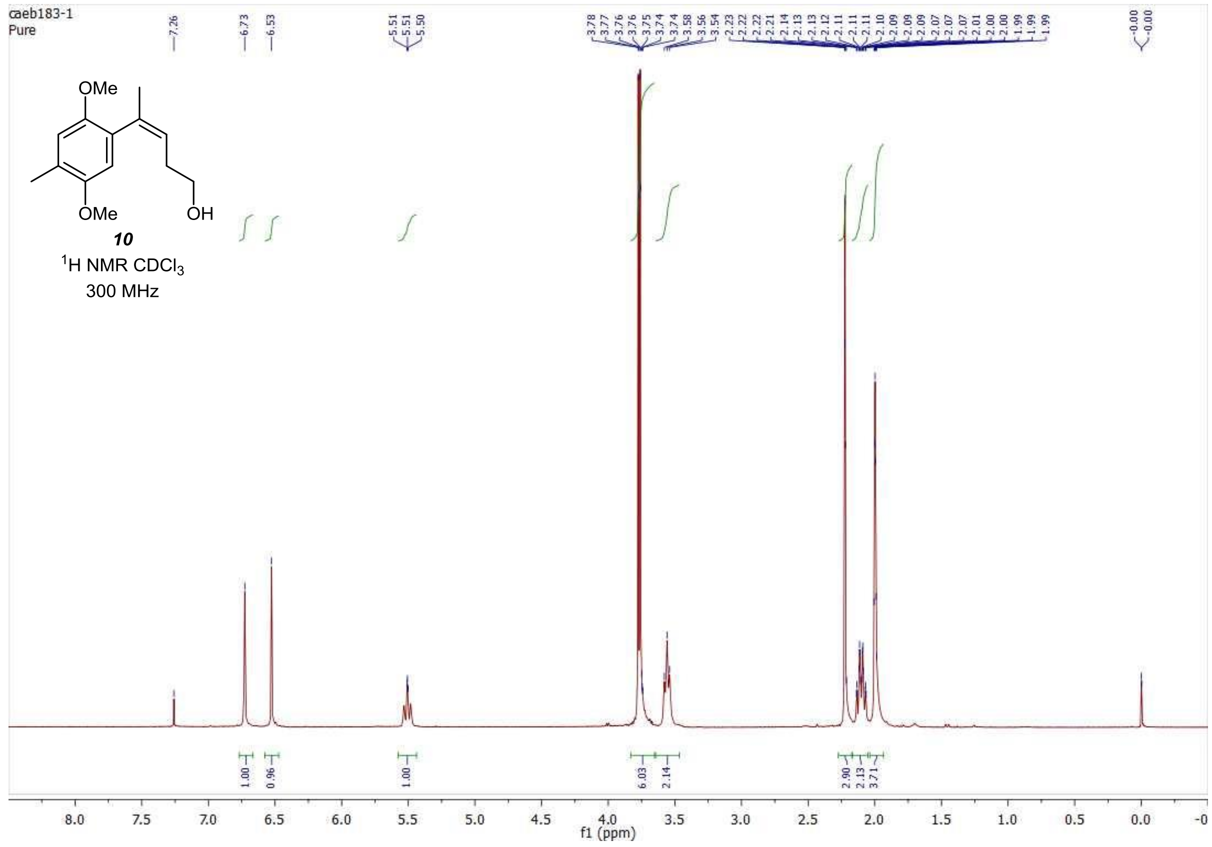
caeb183-1
Pure
OMe
OMe
OH
10
1H NMR CDCl3
300 MHz
f1 (ppm)

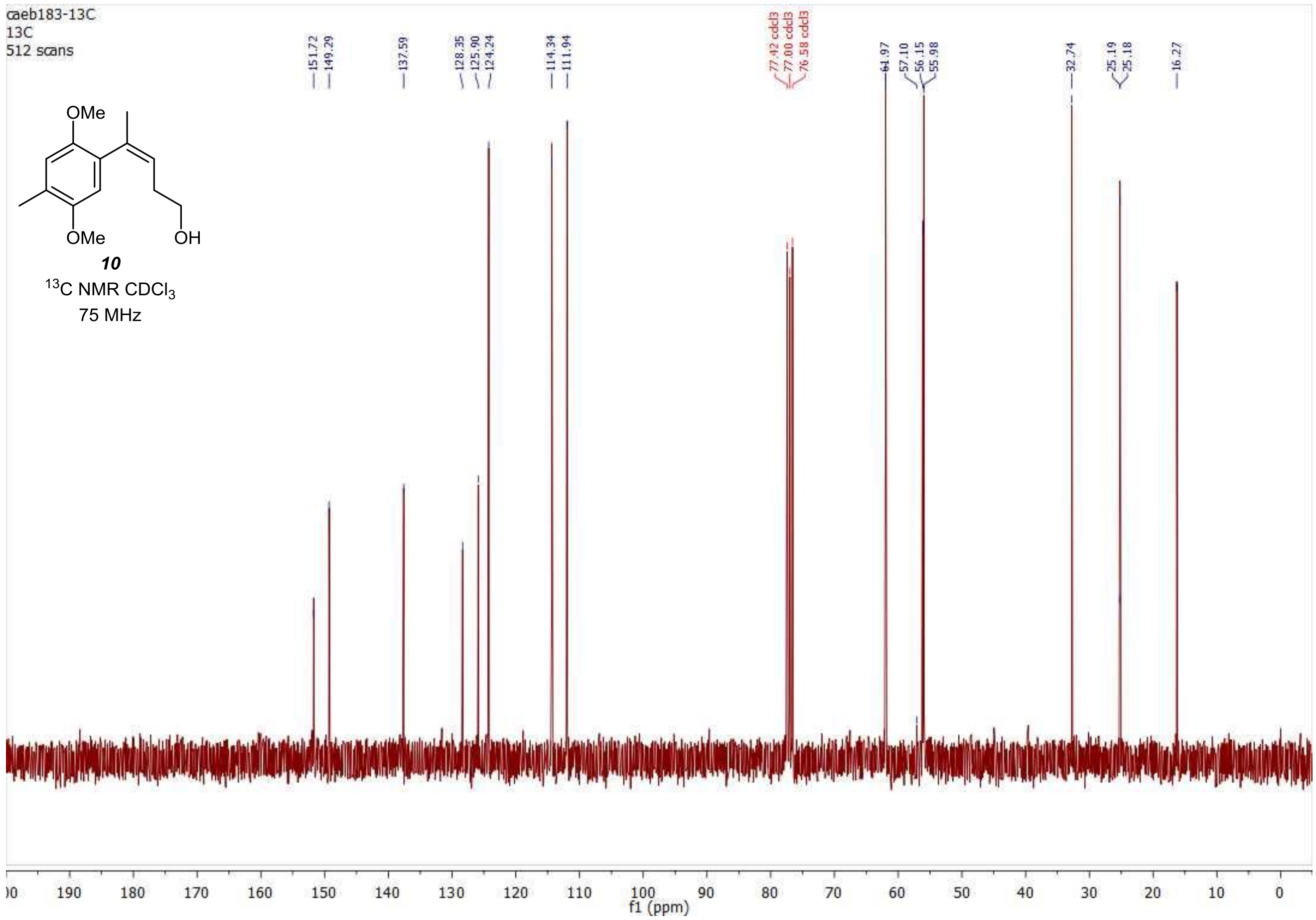
caeb183-13C
13C
512 scans
OMe
OMe
OH
10
13C NMR CDCl3
75 MHz
f1 (ppm)

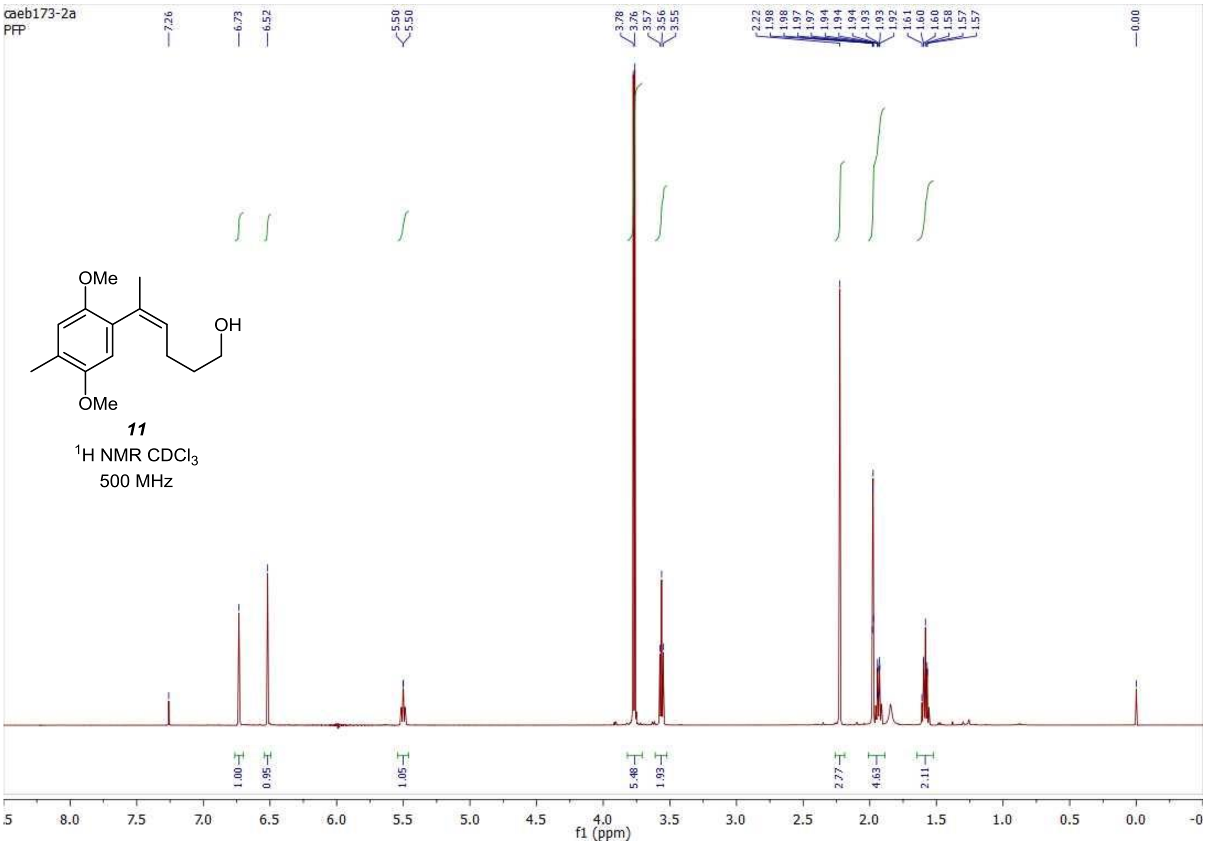
caeb173-2a
PFP
7.26
6.73
6.52
5.50
5.50
3.78
3.76
3.57
3.56
3.55
2.22
1.98
1.98
1.97
1.97
1.94
1.94
1.94
1.93
1.93
1.92
1.61
1.60
1.60
1.58
1.57
1.57
0.00
OMe
OH
OMe
11
¹H NMR CDCl₃
500 MHz
1.00
0.95
1.05
5.48
1.93
2.77
4.63
2.11
f1 (ppm)

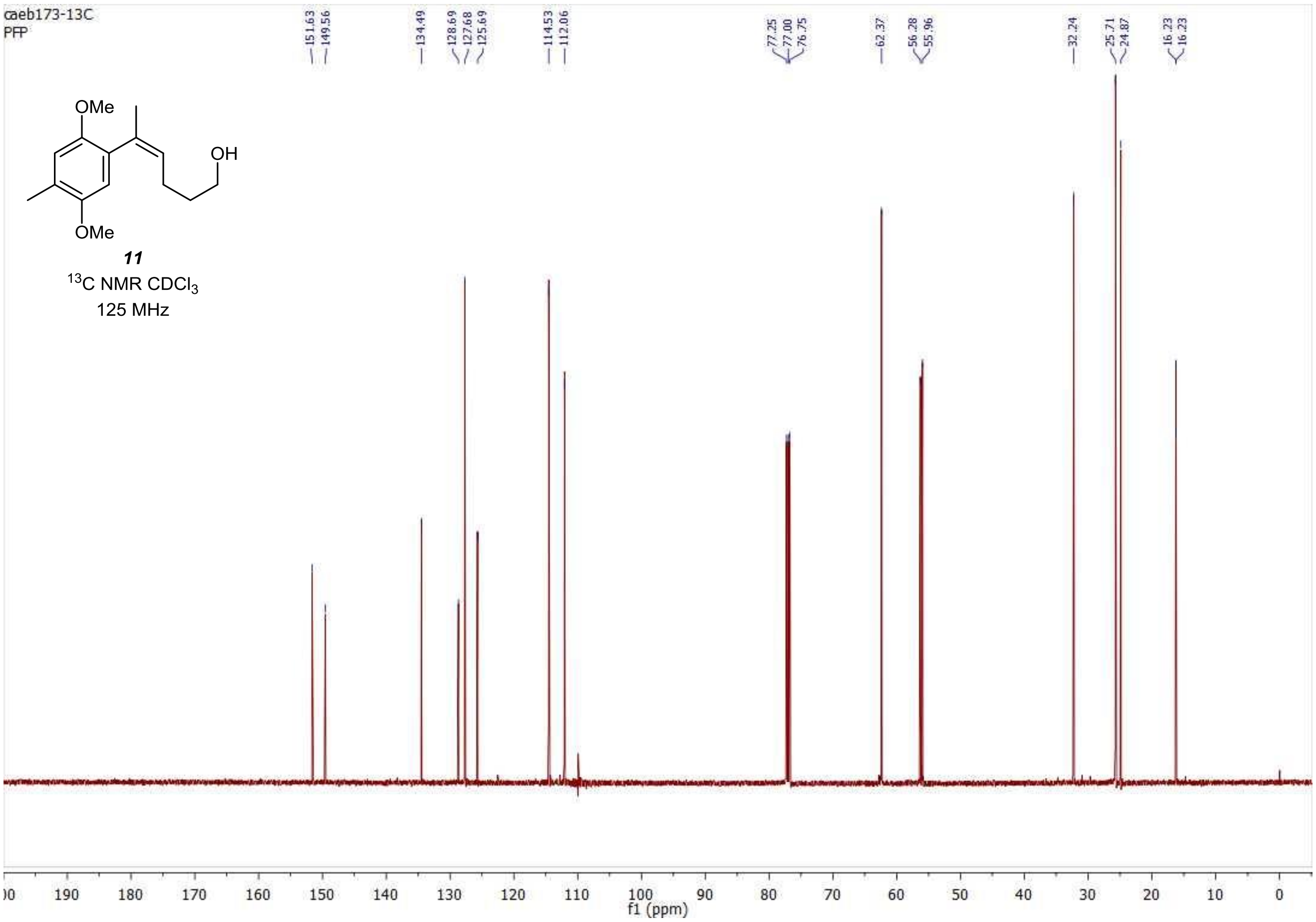
caeb173-13C
PFP
151.63
149.56
134.49
128.69
127.68
125.69
114.53
112.06
77.25
77.00
76.75
62.37
56.28
55.96
32.24
25.71
24.87
16.23
16.23
OMe
OH
OMe
11
¹³C NMR CDCl₃
125 MHz
f1 (ppm)

EDWA087 pure

Sample Name:

Data Collected on:
inovaNMR-inova500
Archive directory:

Sample directory:

FidFile: PROTON

Pulse Sequence: PROTON (s2pul)
Solvent: cdcl3
Data collected on: Sep 5 2008

Operator: ewold800

Relax. delay 1.000 sec
Pulse 45.0 degrees
Acq. time 2.048 sec
Width 8000.0 Hz
16 repetitions
OBSERVE H1, 499.7597864 MHz
DATA PROCESSING
FT size 32768
Total time 0 min 49 sec

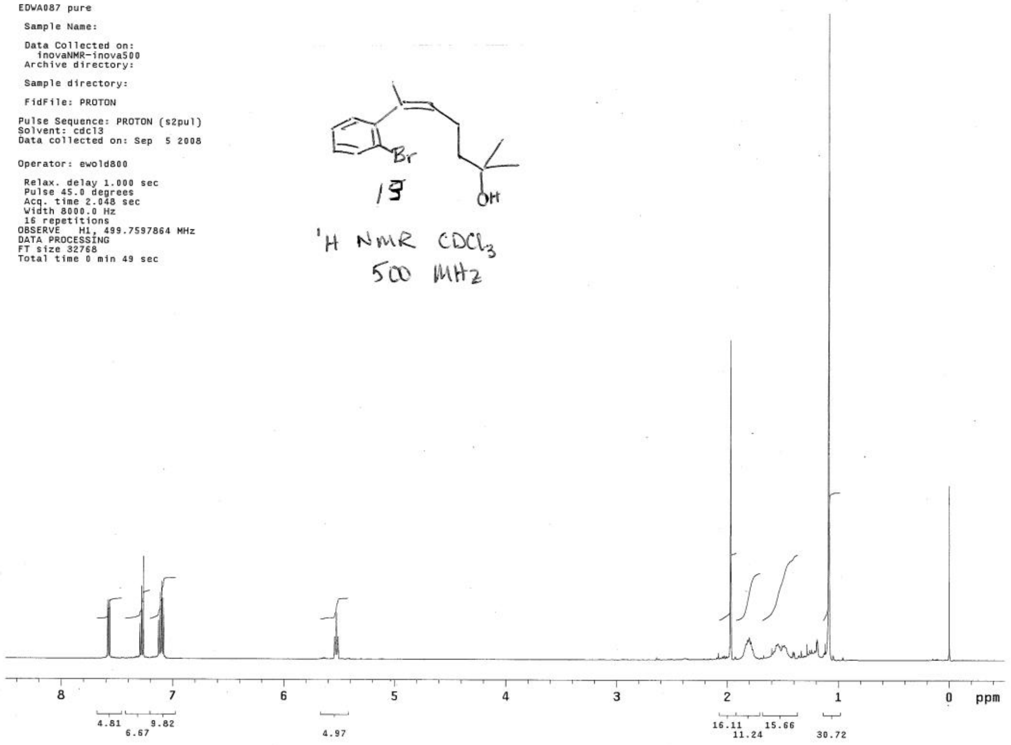

EDWA087 pure

Sample Name:

Data Collected on:
inovaNMR-inova500
Archive directory:

Sample directory:

FidFile: CARBON

Pulse Sequence: CARBON (s2pul)
Solvent: cdcl3
Data collected on: Sep 5 2008

Operator: ewold800

Relax. delay 1.000 sec
Pulse 45.0 degrees
Acq. time 1.042 sec
Width 31446.5 Hz
1000 repetitions
OBSERVE C13, 125.6646979 MHz
DECOUPLE H1, 499.7622837 MHz
Power 39 dB
continuously on
WALTZ-16 modulated
DATA PROCESSING
Line broadening 0.5 Hz
FT size 65536
Total time 34 min

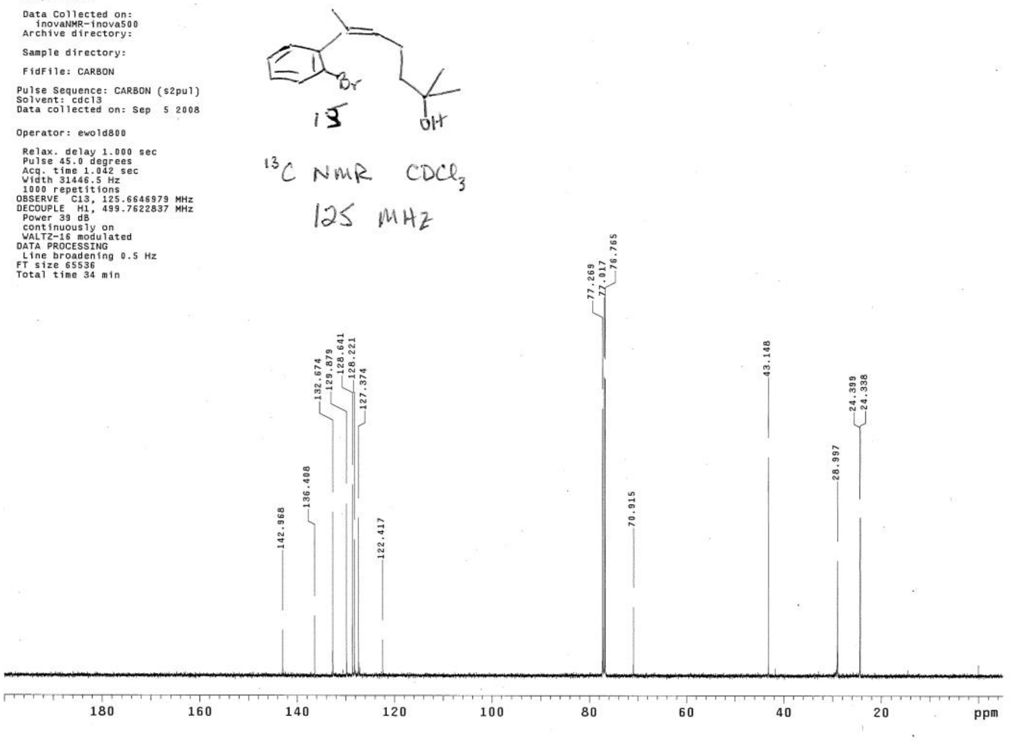

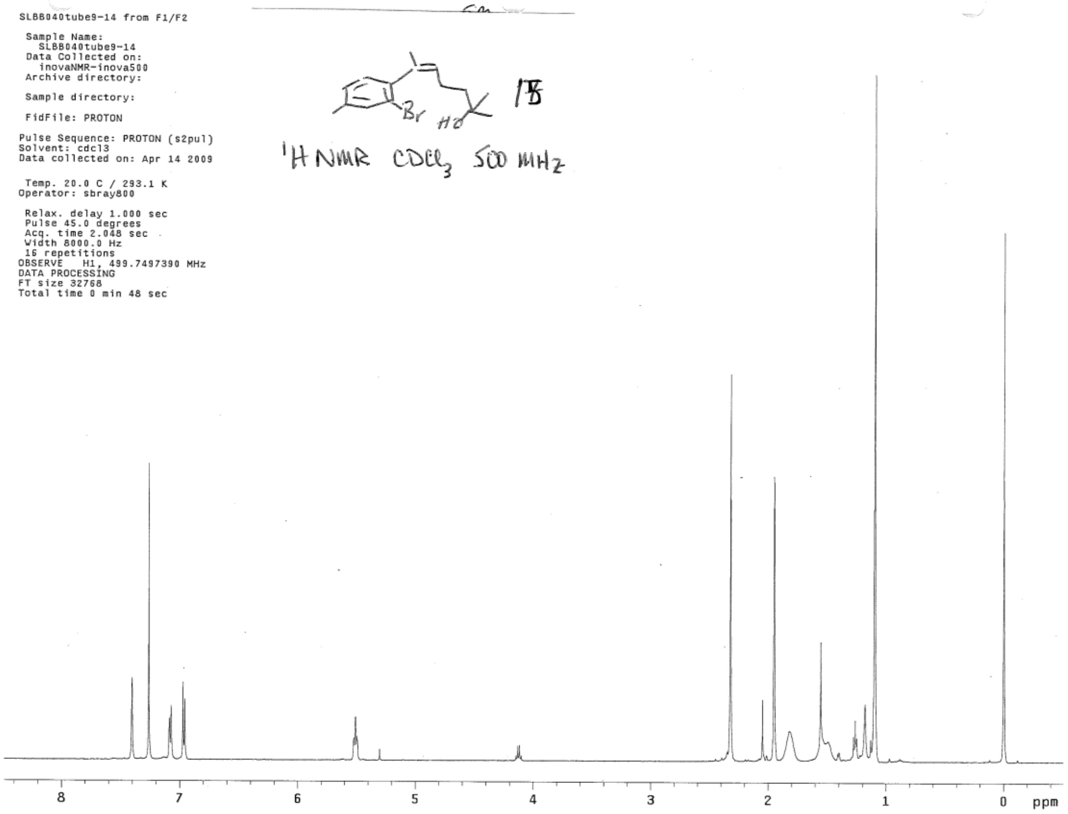
SLBB040tube9-14 from F1/F2
Sample Name:
SLBB040tube9-14
Data Collected on:
inovaNMR-inova500
Archive directory:
Sample directory:
FidFile: PROTON
Pulse Sequence: PROTON (s2pul)
Solvent: cdcl3
Data collected on: Apr 14 2009
Temp. 20.0 C / 293.1 K
Operator: sbray800
Relax. delay 1.000 sec
Pulse 45.0 degrees
Acq. time 2.048 sec
Width 8000.0 Hz
16 repetitions
OBSERVE H1, 499.7497390 MHz
DATA PROCESSING
FT size 32768
Total time 0 min 48 sec
Br
HO
15
1H NMR CDCl3 500 MHz
8
7
6
5
4
3
2
1
0
ppm

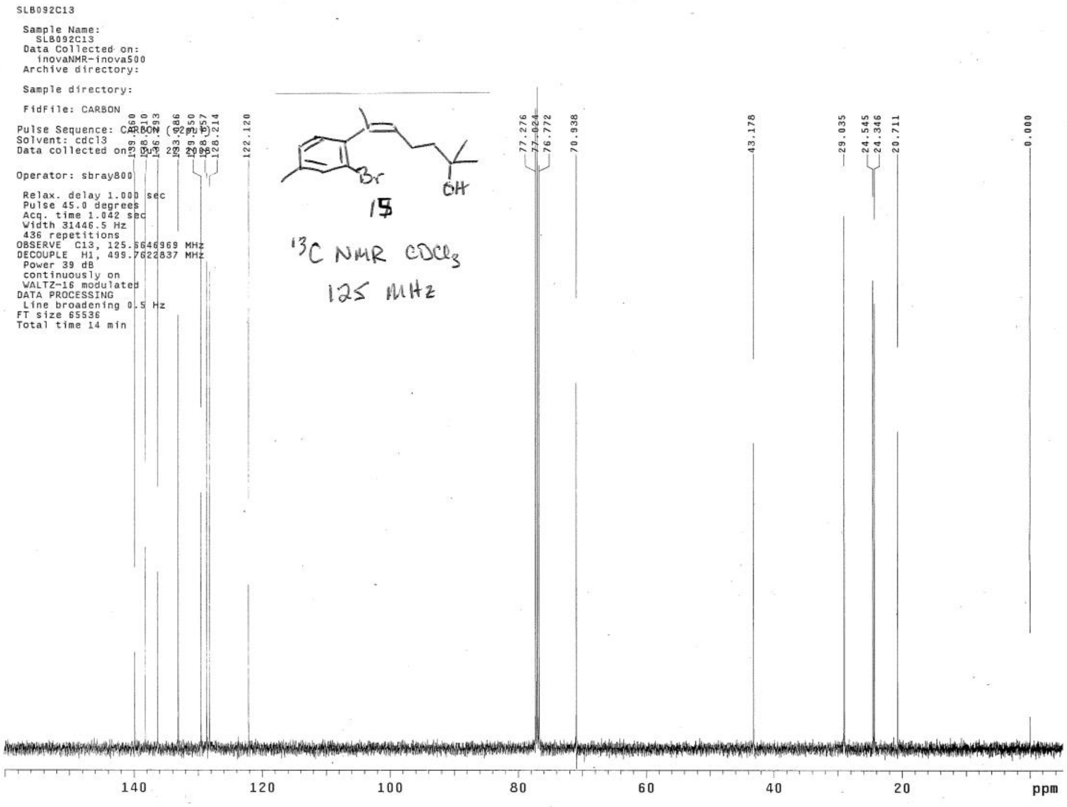
SLB092C13
Sample Name:
SLB092C13
Data Collected on:
inovaNMR-inova500
Archive directory:
Sample directory:
FidFile: CARBON
Solvent: cdcl3
Operator: sbray800
Relax. delay 1.000 sec
Pulse 45.0 degrees
Acq. time 1.042 sec
Width 31446.5 Hz
436 repetitions
OBSERVE C13, 125.5646969 MHz
DECOUPLE H1, 499.7622837 MHz
Power 39 dB
continuously on
WALTZ-16 modulated
DATA PROCESSING
Line broadening 0.5 Hz
FT size 65536
Total time 14 min
128.214
122.120
77.276
77.024
76.772
70.938
43.178
29.035
24.545
24.346
20.711
0.000
Br
OH
15
13C NMR CDCl3
125 MHz
140
120
100
80
60
40
20
ppm

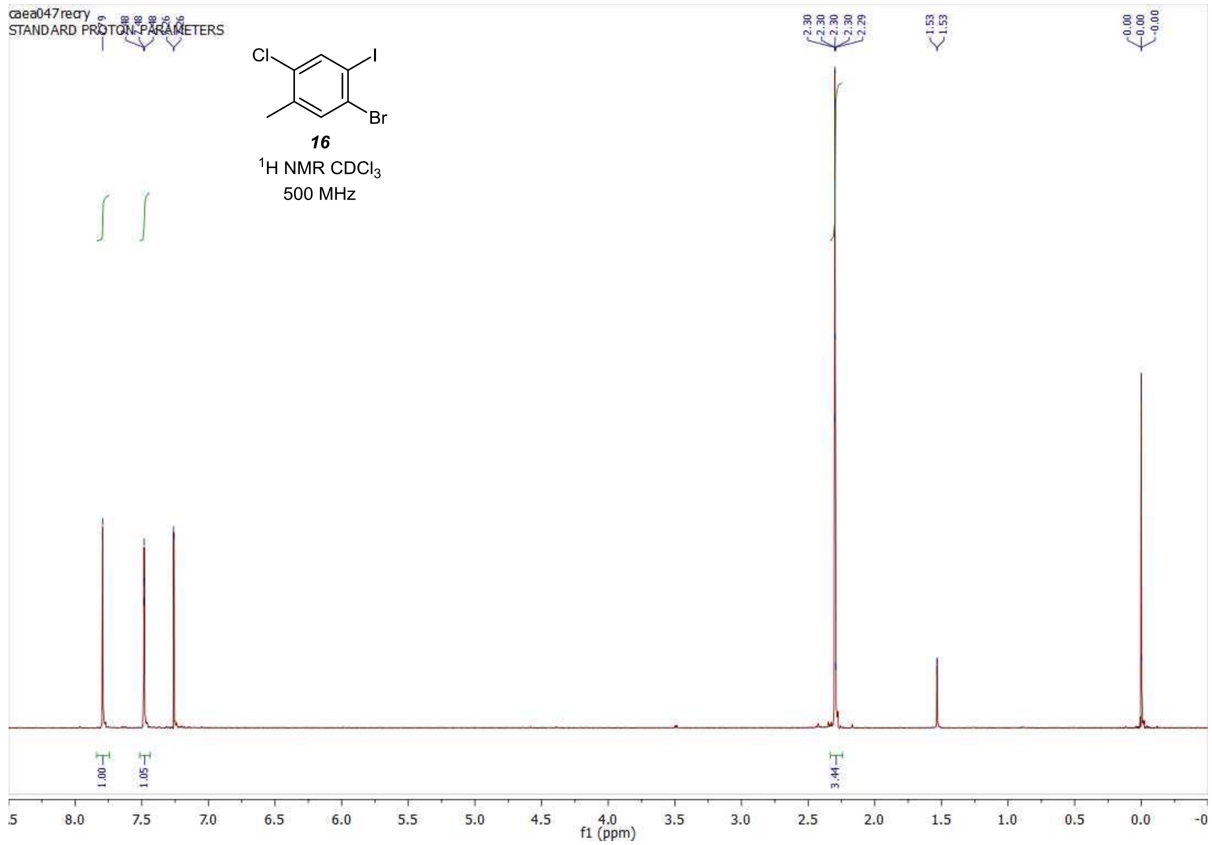
caea047recry
STANDARD PROTON PARAMETERS
2.30
2.30
2.30
2.30
2.29
1.53
1.53
0.00
0.00
-0.00
Cl
I
Br
16
1H NMR CDCl3
500 MHz
1.00
1.05
3.44
f1 (ppm)

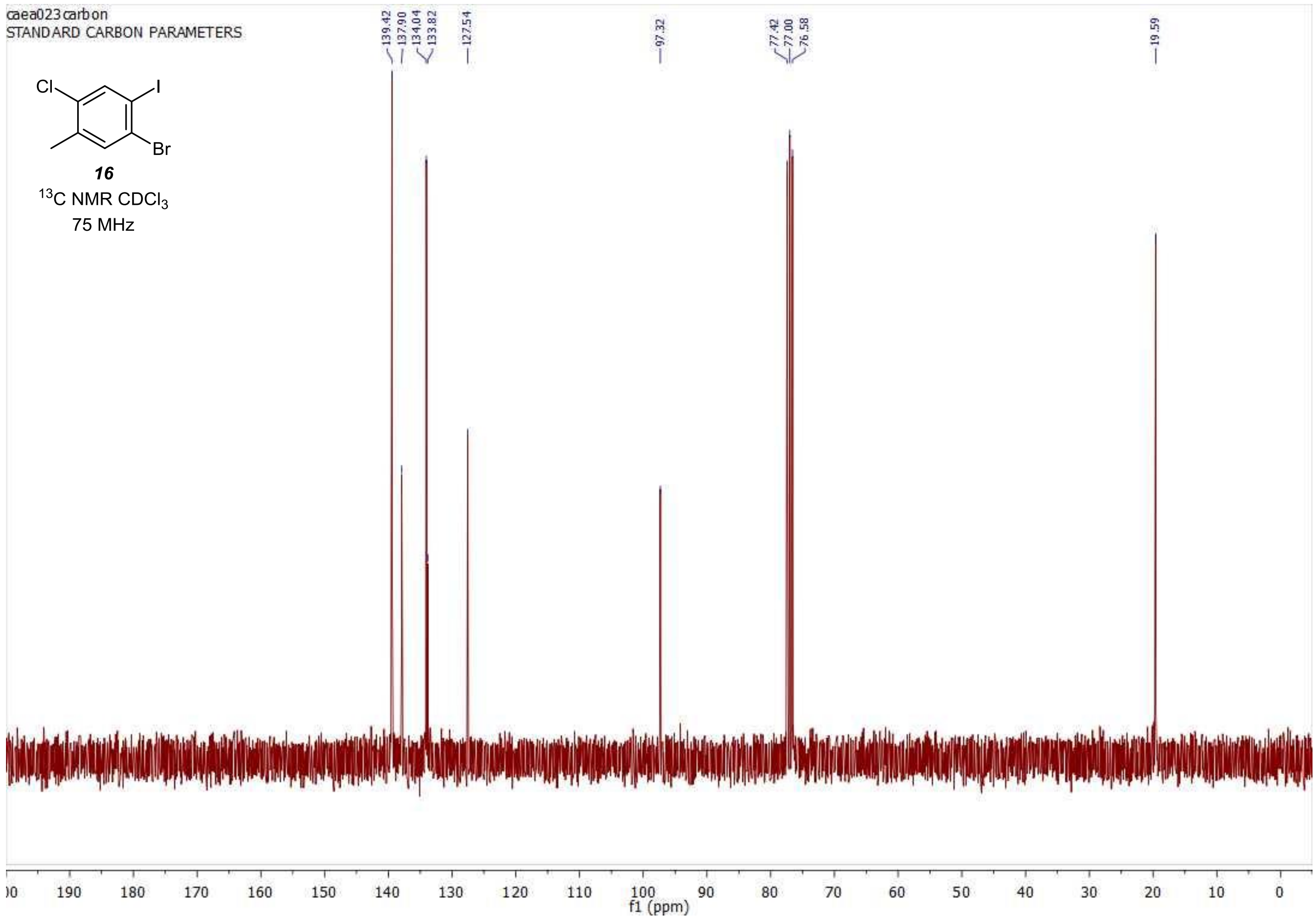
caea023carbon
STANDARD CARBON PARAMETERS
139.42
137.90
134.04
133.82
127.54
97.32
77.42
77.00
76.58
19.59
Cl
I
Br
16
13C NMR CDCl3
75 MHz
f1 (ppm)

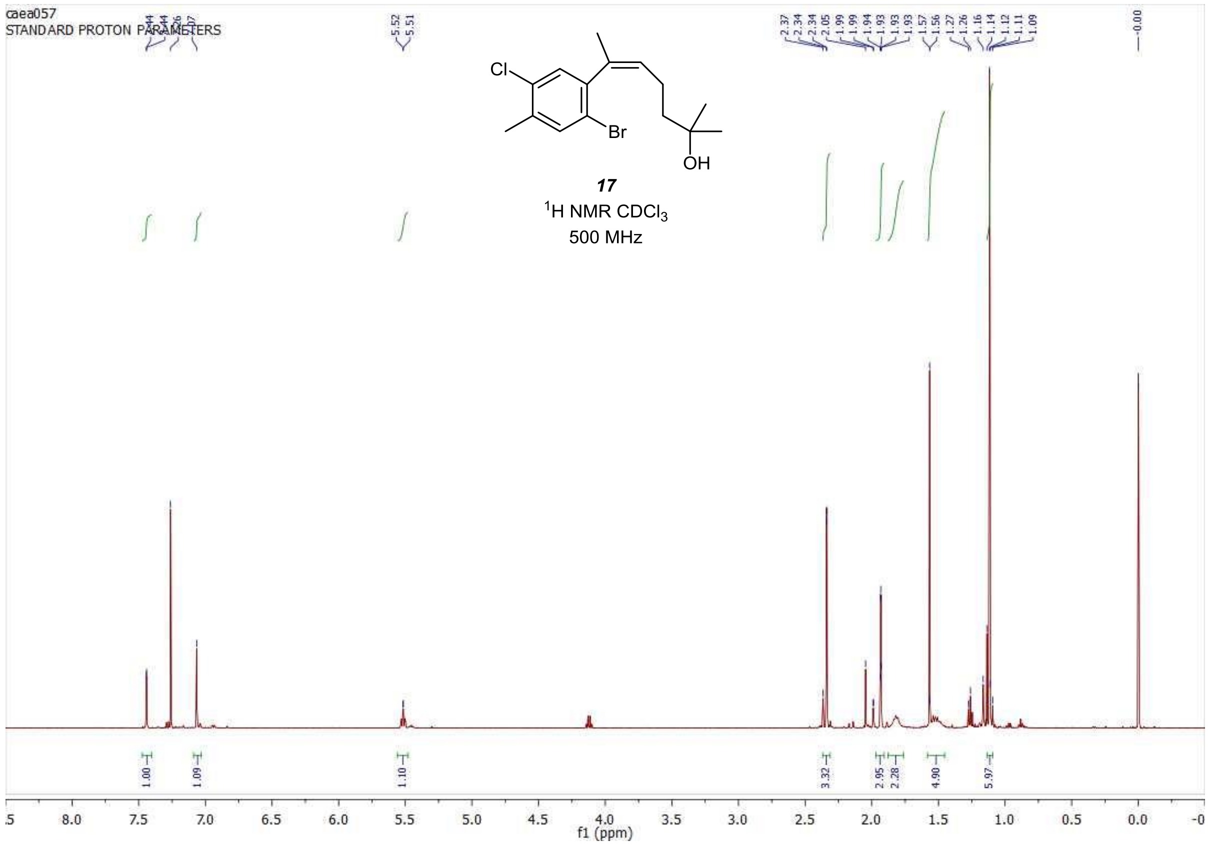
caea057
STANDARD PROTON PARAMETERS
17
¹H NMR CDCl3
500 MHz
f1 (ppm)

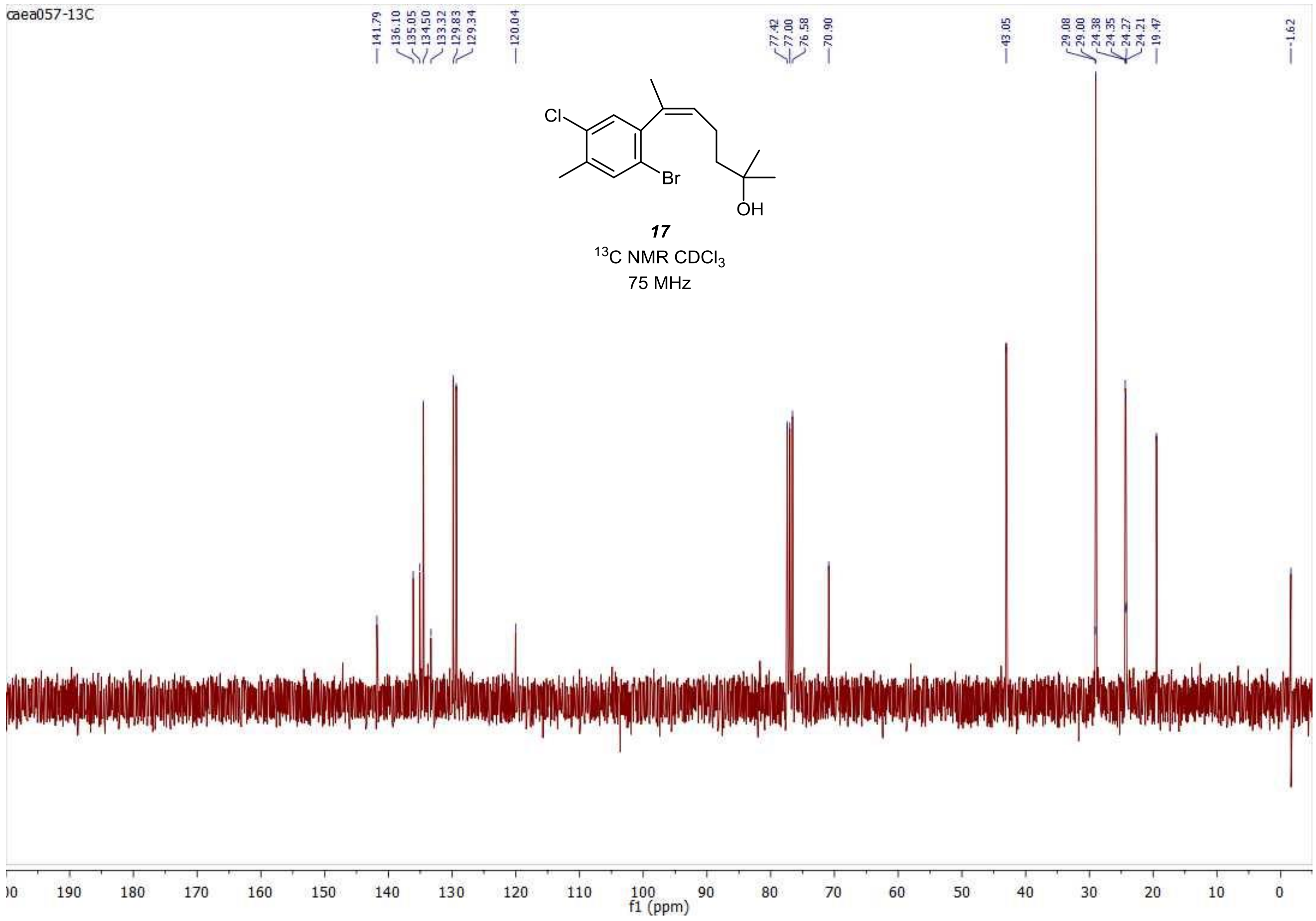
caea057-13C
17
¹³C NMR CDCl3
75 MHz
f1 (ppm)

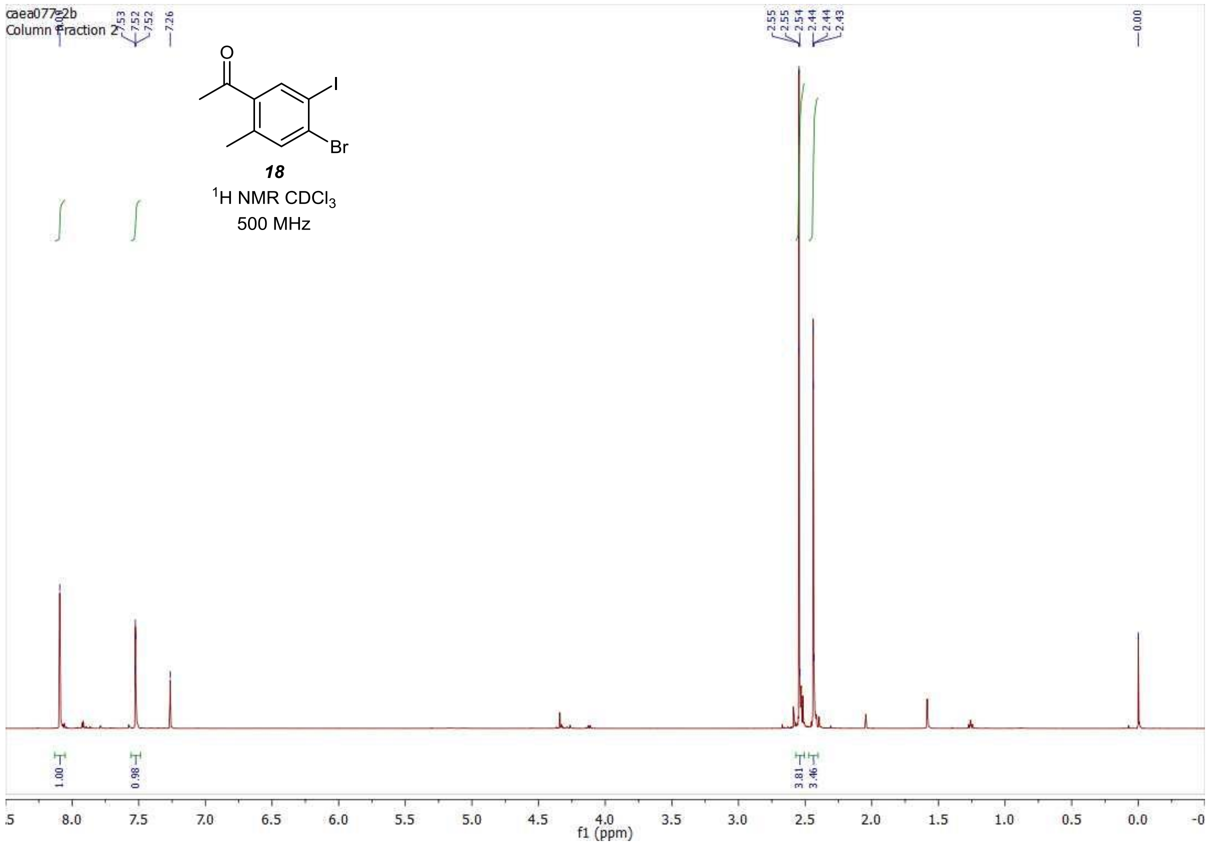
caea077-2b
Column Fraction 2
8.09
7.53
7.52
7.52
7.26
2.55
2.55
2.54
2.44
2.44
2.43
0.00
O
I
Br
18
^{1}H NMR $CDCl_3$
500 MHz
1.00
0.98
3.81
3.46
f1 (ppm)

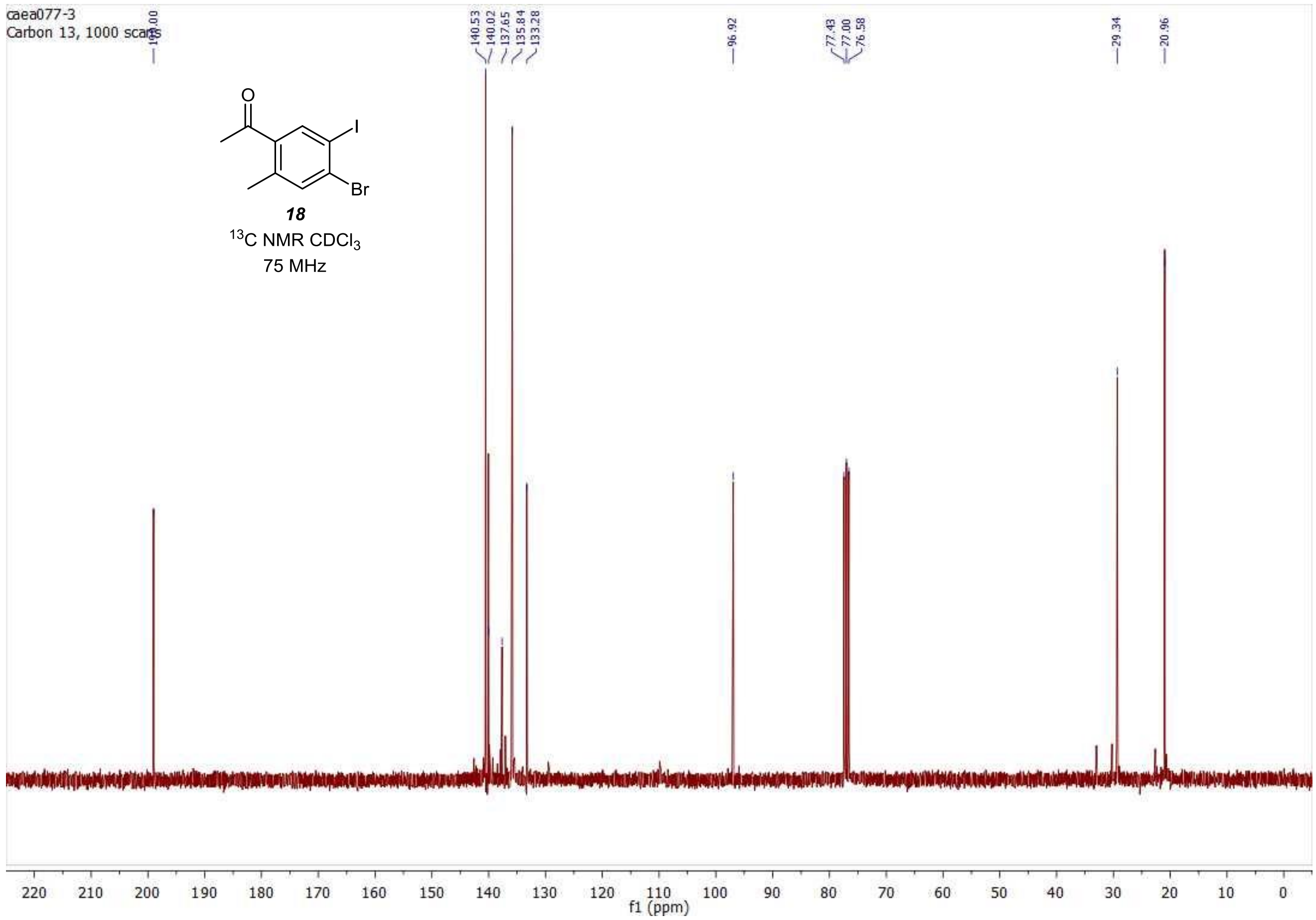
caea077-3
Carbon 13, 1000 scans
199.00
140.53
140.02
137.65
135.84
133.28
96.92
77.43
77.00
76.58
29.34
20.96
O
I
Br
18
^{13}C NMR $CDCl_3$
75 MHz
f1 (ppm)

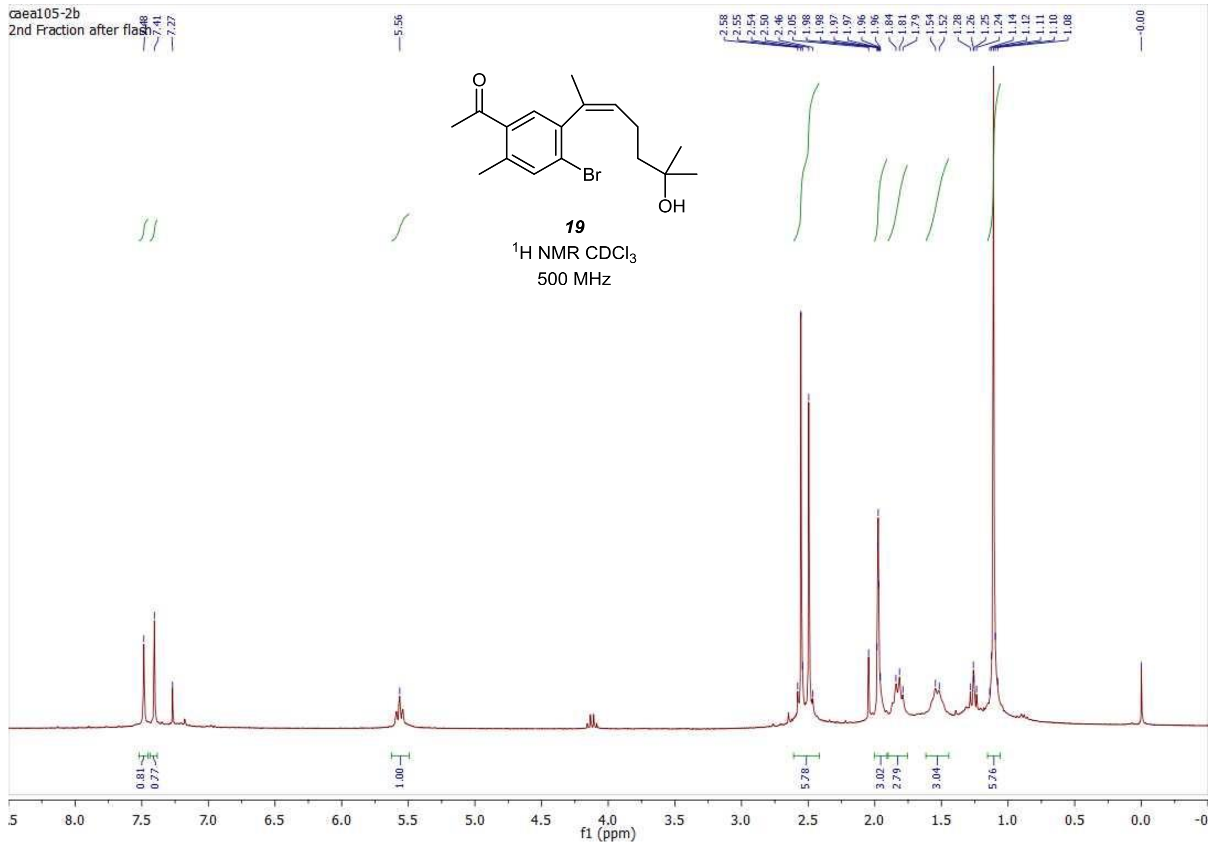
caea105-2b
2nd Fraction after flash
Br
OH
19
^{1}H NMR CDCl$_3$
500 MHz
f1 (ppm)

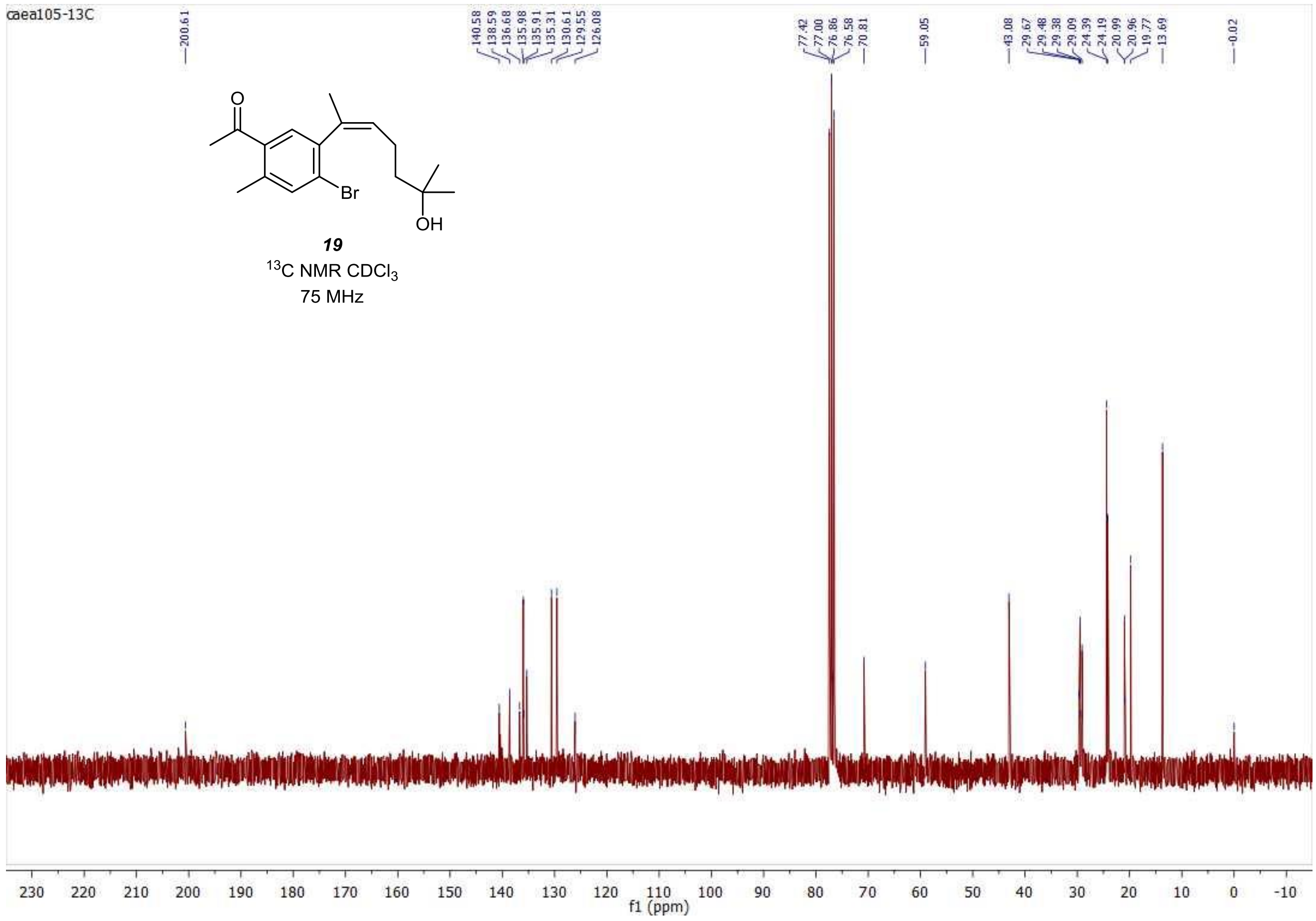
caea105-13C
Br
OH
19
^{13}C NMR CDCl$_3$
75 MHz
f1 (ppm)

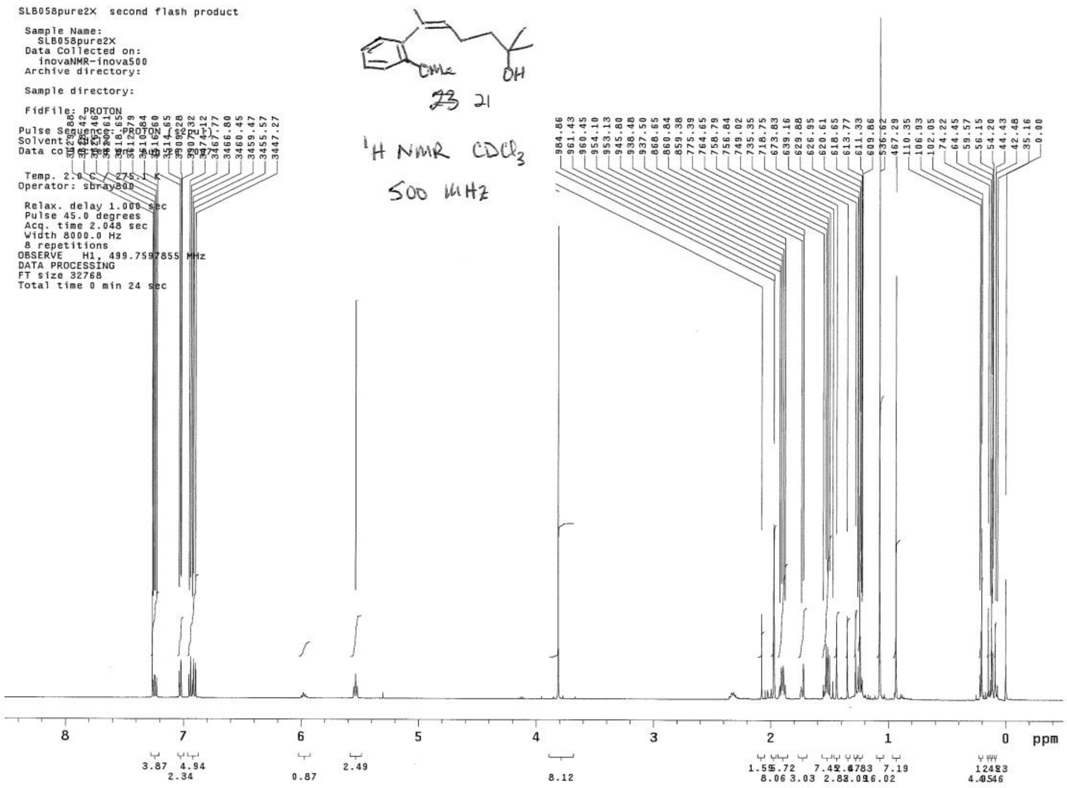
SLB058pure2X second flash product
Sample Name:
SLB058pure2X
Data Collected on:
inovaNMR-inova500
Archive directory:
Sample directory:
FidFile: PROTON
Pulse Sequence: PROTON (s2pul)
Temp. 2.0 C / 275.1 K
Operator: sbray800
Relax. delay 1.000 sec
Pulse 45.0 degrees
Acq. time 2.048 sec
Width 8000.0 Hz
8 repetitions
OBSERVE H1, 499.7597855 MHz
DATA PROCESSING
FT size 32768
Total time 0 min 24 sec
OMe
OH
21
1H NMR CDCl3
500 MHz
ppm

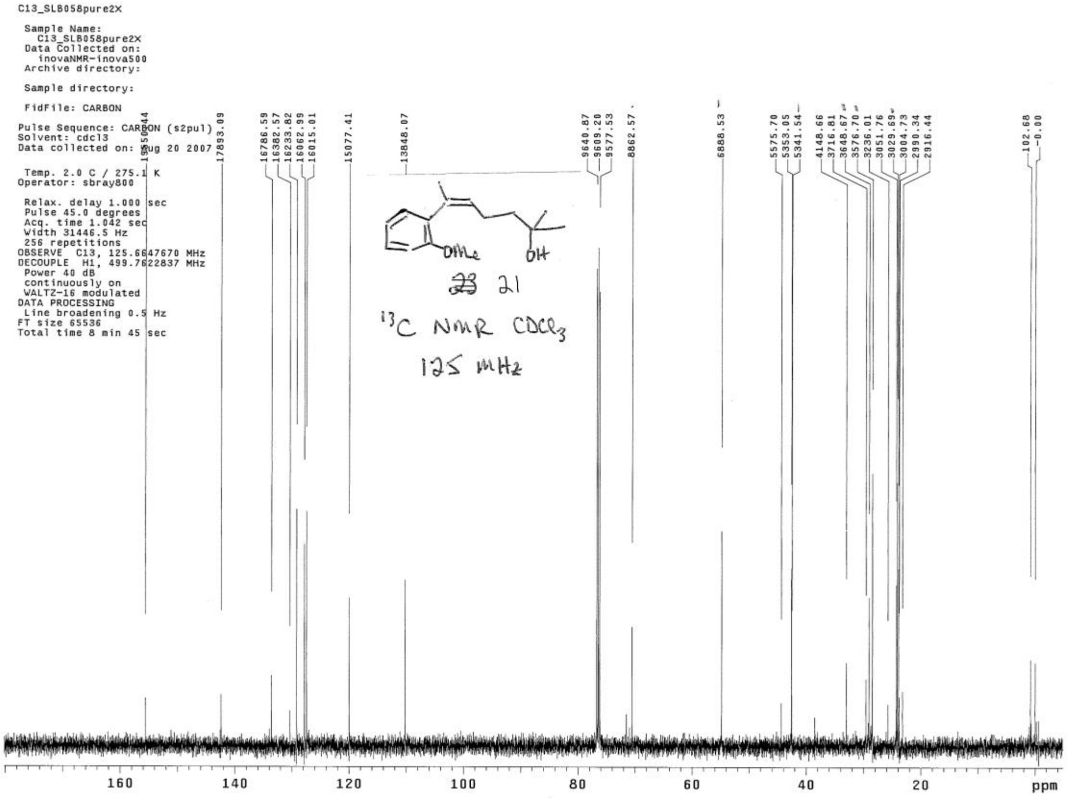
C13_SLB058pure2X
Sample Name:
C13_SLB058pure2X
Data Collected on:
inovaNMR-inova500
Archive directory:
Sample directory:
FidFile: CARBON
Pulse Sequence: CARBON (s2pul)
Solvent: cdcl3
Data collected on: Aug 20 2007
Temp. 2.0 C / 275.1 K
Operator: sbray800
Relax. delay 1.000 sec
Pulse 45.0 degrees
Acq. time 1.042 sec
Width 31446.5 Hz
256 repetitions
OBSERVE C13, 125.6647670 MHz
DECOUPLE H1, 499.7622837 MHz
Power 40 dB
continuously on
WALTZ-16 modulated
DATA PROCESSING
Line broadening 0.5 Hz
FT size 65536
Total time 8 min 45 sec
OMe
OH
21
13C NMR CDCl3
125 MHz
ppm

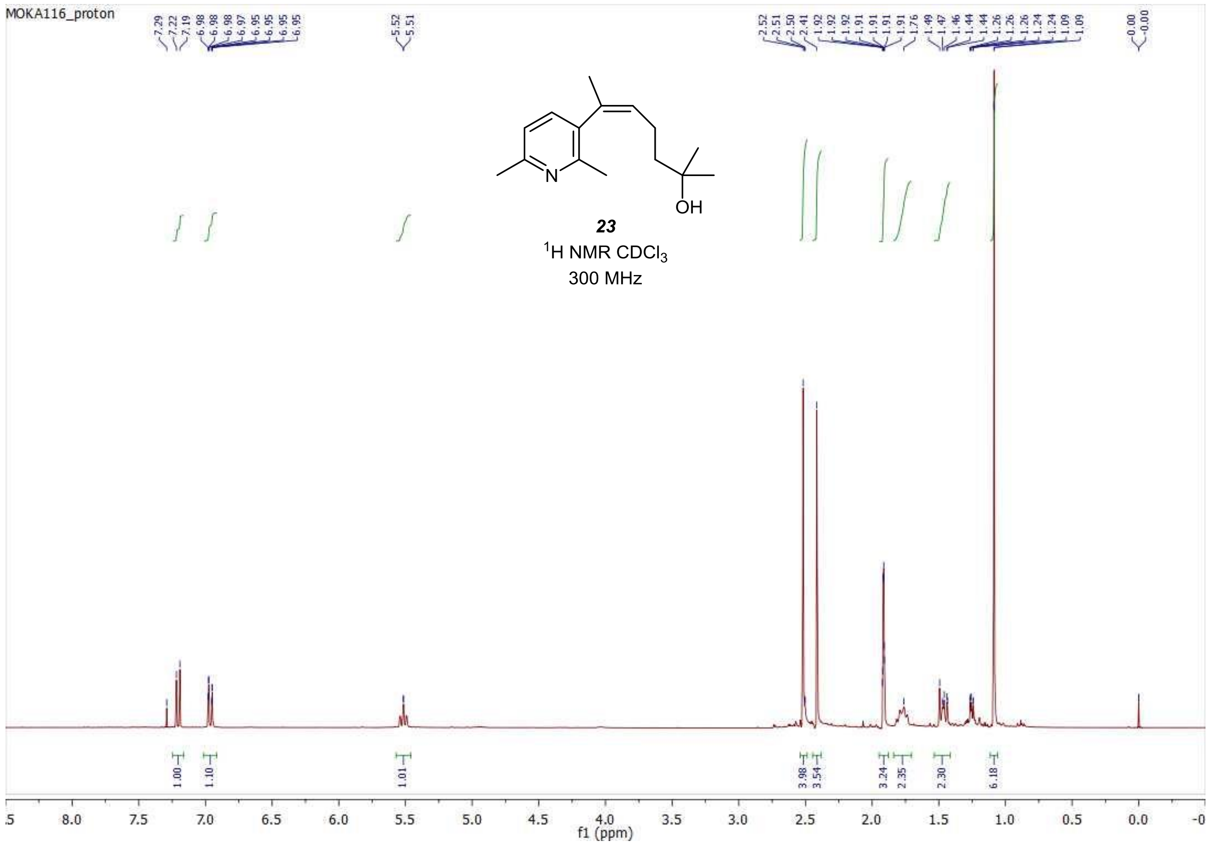
MOKA116_proton
N
OH
23
^{1}H NMR $CDCl_3$
300 MHz
f1 (ppm)

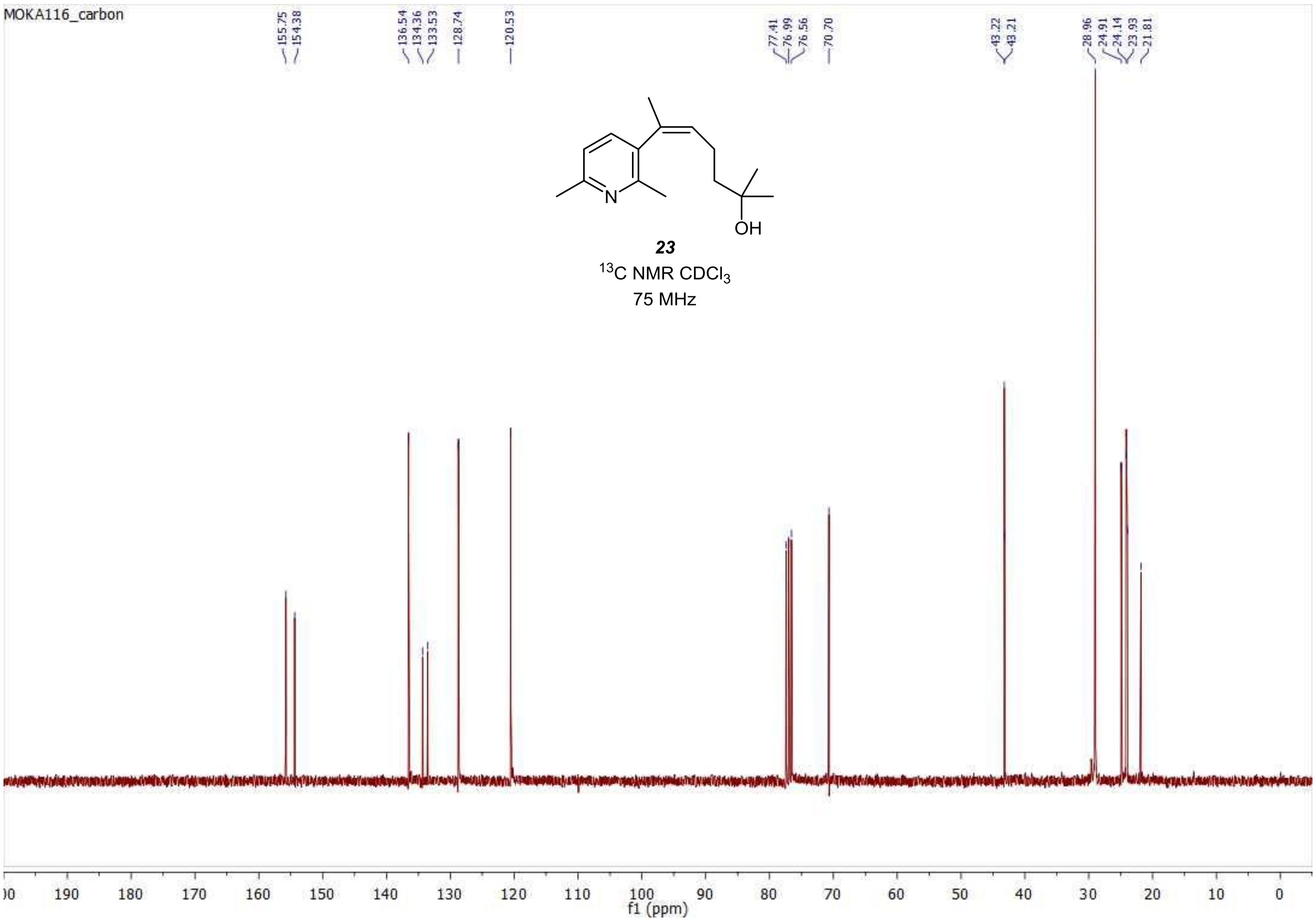
MOKA116_carbon
N
OH
23
^{13}C NMR $CDCl_3$
75 MHz
f1 (ppm)

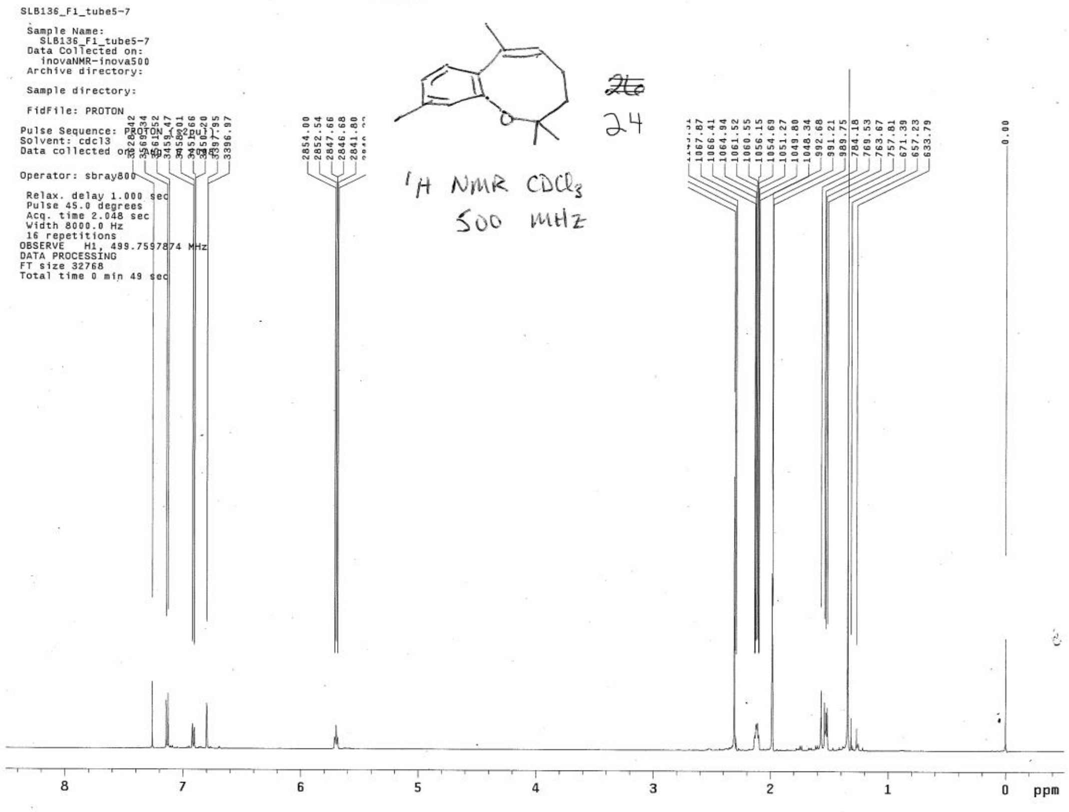
SLB136_F1_tube5-7
Sample Name:
SLB136_F1_tube5-7
Data Collected on:
inovaNMR-inova500
Archive directory:
Sample directory:
FidFile: PROTON
Pulse Sequence: PROTON (s2pul)
Solvent: cdcl3
Operator: sbray800
Relax. delay 1.000 sec
Pulse 45.0 degrees
Acq. time 2.048 sec
Width 8000.0 Hz
16 repetitions
OBSERVE H1, 499.7597874 MHz
DATA PROCESSING
FT size 32768
Total time 0 min 49 sec
24
1H NMR CDCl3
500 MHz
8 7 6 5 4 3 2 1 0 ppm

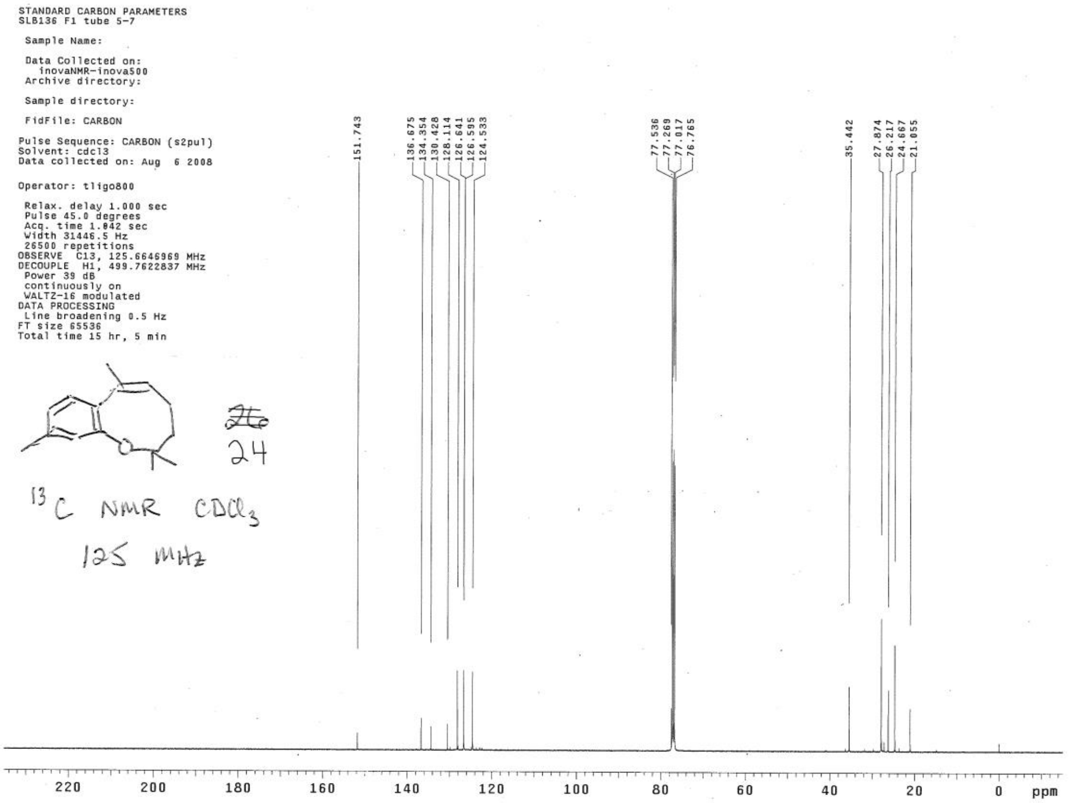
STANDARD CARBON PARAMETERS
SLB136 F1 tube 5-7
Sample Name:
Data Collected on:
inovaNMR-inova500
Archive directory:
Sample directory:
FidFile: CARBON
Pulse Sequence: CARBON (s2pul)
Solvent: cdcl3
Data collected on: Aug 6 2008
Operator: tligo800
Relax. delay 1.000 sec
Pulse 45.0 degrees
Acq. time 1.042 sec
Width 31446.5 Hz
26500 repetitions
OBSERVE C13, 125.6646969 MHz
DECOUPLE H1, 499.7622837 MHz
Power 39 dB
continuously on
WALTZ-16 modulated
DATA PROCESSING
Line broadening 0.5 Hz
FT size 65536
Total time 15 hr, 5 min
151.743
136.675
134.354
130.428
128.114
126.641
126.595
124.533
77.536
77.269
77.017
76.765
35.442
27.874
26.217
24.667
21.055
24
13C NMR CDCl3
125 MHz
220 200 180 160 140 120 100 80 60 40 20 0 ppm

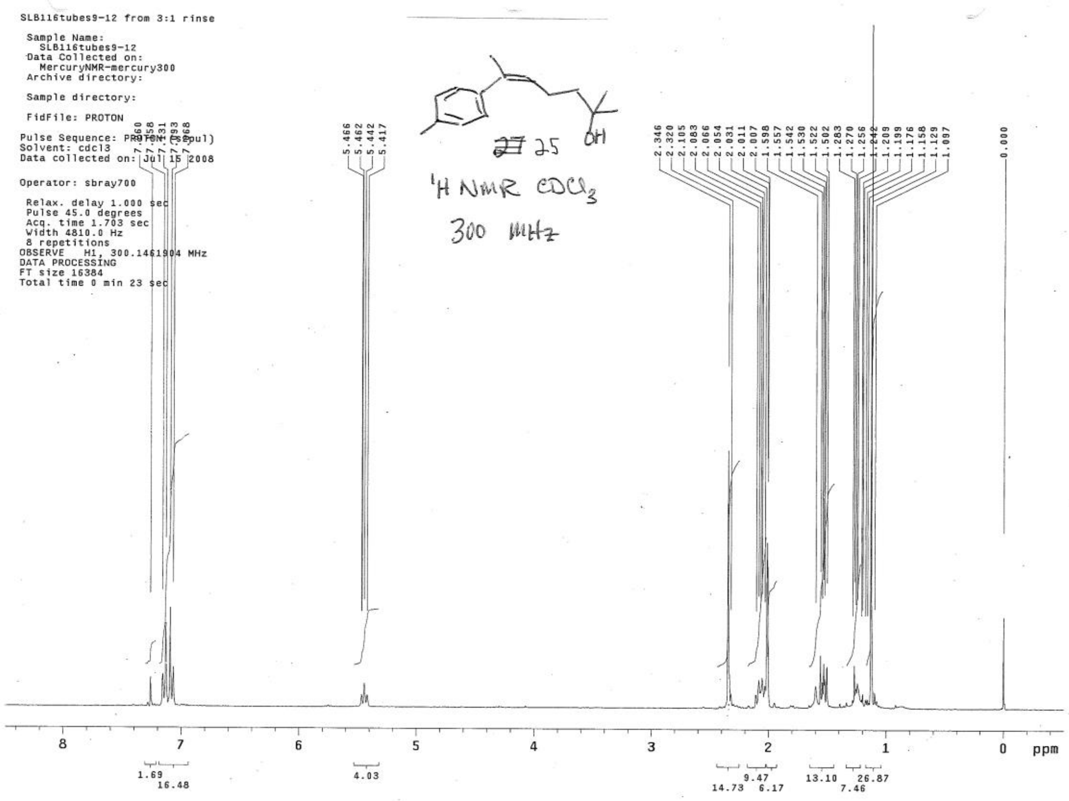
SLB116tubes9-12 from 3:1 rinse
Sample Name:
SLB116tubes9-12
Data Collected on:
MercuryNMR-mercury300
Archive directory:
Sample directory:
FidFile: PROTON
Pulse Sequence: PROTON (s2pul)
Solvent: cdcl3
Data collected on: Jul 15 2008
Operator: sbray700
Relax. delay 1.000 sec
Pulse 45.0 degrees
Acq. time 1.703 sec
Width 4810.0 Hz
8 repetitions
OBSERVE H1, 300.1461904 MHz
DATA PROCESSING
FT size 16384
Total time 0 min 23 sec
27 25
¹H NMR CDCl₃
300 MHz
ppm

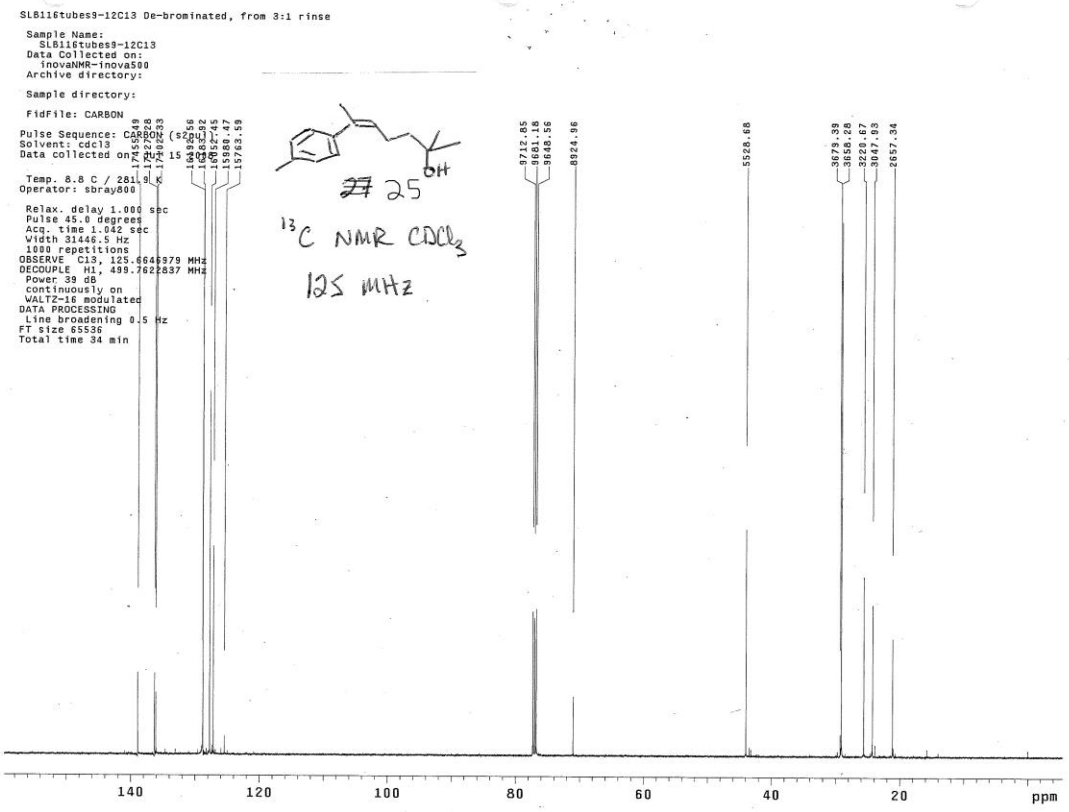
SLB116tubes9-12C13 De-brominated, from 3:1 rinse
Sample Name:
SLB116tubes9-12C13
Data Collected on:
inovaNMR-inova500
Archive directory:
Sample directory:
FidFile: CARBON
Pulse Sequence: CARBON (s2pul)
Solvent: cdcl3
Temp. 8.8 C / 281.9 K
Operator: sbray800
Relax. delay 1.000 sec
Pulse 45.0 degrees
Acq. time 1.042 sec
Width 31446.5 Hz
1000 repetitions
OBSERVE C13, 125.6646979 MHz
DECOUPLE H1, 499.7622837 MHz
Power 39 dB
continuously on
WALTZ-16 modulated
DATA PROCESSING
Line broadening 0.5 Hz
FT size 65536
Total time 34 min
27 25
¹³C NMR CDCl₃
125 MHz
ppm

SLBB036tube5-7

Sample Name:
SLBB036tube5-7
Data Collected on:
inovaNMR-inova500
Archive directory:

Sample directory:

FidFile: PROTON

Pulse Sequence: PROTON (s2pul)
Solvent: cdcl3
Data collected on: Feb 5 2009

Temp. 20.0 C / 293.1 K
Operator: sbray800

Relax. delay 1.000 sec
Pulse 45.0 degrees
Acq. time 2.048 sec
Width 8000.0 Hz
16 repetitions
OBSERVE H1, 499.7497394 MHz
DATA PROCESSING
FT size 32768
Total time 0 min 49 sec

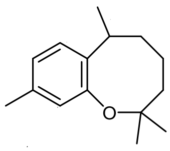

26

^{1}H NMR $CDCl_3$

500 MHz

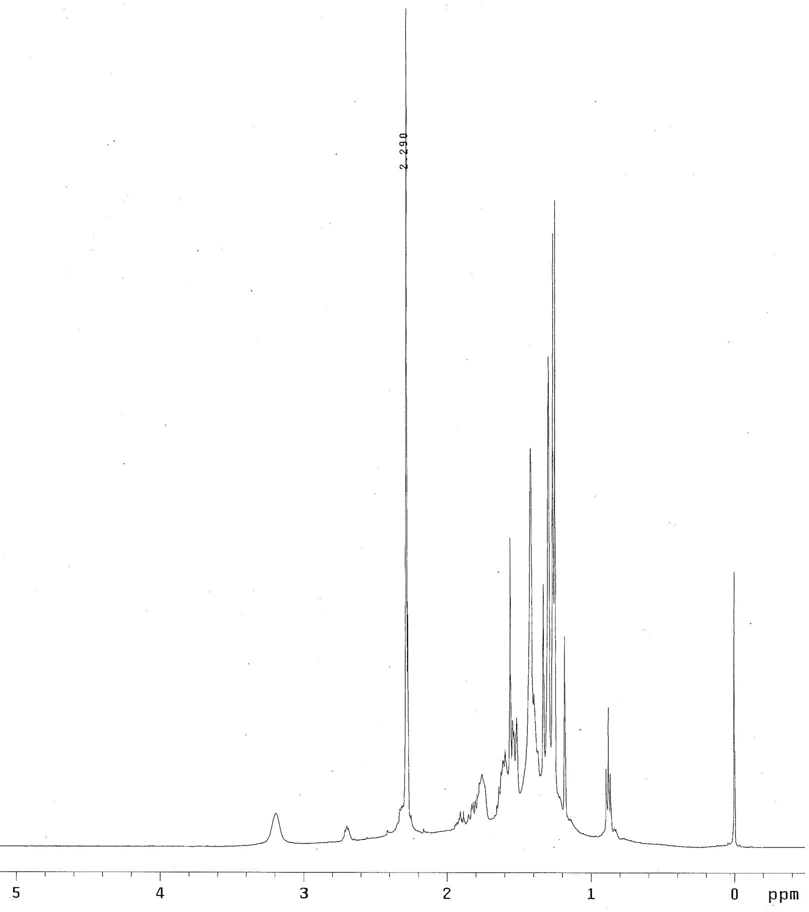

Supplement: File 1 — Full experimental details and copies of 1H and 13C NMR spectra for all new compounds. [file Beilstein_J_Org_Chem-13-2122-s001.pdf]
